# Protein co-migration database (PCoM -DB) for *Arabidopsis* thylakoids and *Synechocystis* cells

**DOI:** 10.1186/2193-1801-2-148

**Published:** 2013-04-08

**Authors:** Atsushi Takabayashi, Ryosuke Kadoya, Masayoshi Kuwano, Katsunori Kurihara, Hisashi Ito, Ryouichi Tanaka, Ayumi Tanaka

**Affiliations:** 1 Institute of Low Temperature Science, Hokkaido University, N19 W8 Kita-Ku, Sapporo, 060-0819 Japan; 2 Japan Core Research for Evolutionary Science and Technology (CREST), Sapporo, Japan

**Keywords:** Protein complex, Proteome, *Arabidopsis thaliana*, *Synechocystis* sp. PCC 6803, Database

## Abstract

Protein-protein interactions are critical for most cellular processes; however, many remain to be identified. Here, to comprehensively identify protein complexes in photosynthetic organisms, we applied the recently developed approach of blue native PAGE (BN-PAGE) coupled with LC-MS/MS to the thylakoid proteins of *Arabidopsis thaliana* and the whole cell proteins of whole cell proteins of *Synechocystis* sp. PCC 6803. We identified 245 proteins from the purified *Arabidopsis* thylakoid membranes and 1,458 proteins from the whole cells of *Synechocystis* using the method. Next, we generated protein migration profiles that were assessed by plotting the label-free estimations of protein abundances versus migration distance in BN-PAGE. Comparisons between the migration profiles of the major photosynthetic complexes and their band patterns showed that the protein migration profiles were well correlated. Thus, the protein migration profiles allowed us to estimate the molecular size of each protein complex and to identify co-migrated proteins with the proteins of interest by determining the protein pairs that contained peaks in the same gel slice. Finally, we built the protein co-migration database for photosynthetic organisms (PCoM-DB: http://pcomdb.lowtem.hokudai.ac.jp/proteins/top) to make our data publicly accessible online, which stores the analyzed data with a user-friendly interface to compare the migration profiles of proteins of interest. It helps users to find unidentified protein complexes in *Arabidopsis* thylakoids and *Synechocystis* cells. The accumulation of the data from the BN-PAGE coupled with LC-MS/MS should reveal unidentified protein complexes and should aid in understanding the adaptation and the evolution of photosynthetic organisms.

**Electronic supplementary material:**

The online version of this article (doi:10.1186/2193-1801-2-148) contains supplementary material, which is available to authorized users.

## Background

Cellular processes are not only performed by the coordinated activities of individual proteins, but they are also executed by a wide variety of protein complexes. Consequently, the identification of protein complexes is important for understanding protein structure and function and also for understanding dynamic cellular processes. For photosynthetic organisms, photosynthetic cells are where photosynthesis and many other metabolic pathways are highly inter-connected via metabolic products. In addition, light harvesting systems of photosynthetic organisms, which are complexes of pigments and proteins, have been dynamically changed during evolution (Neilson and Durnford 2010). Even among cyanobacteria, the light harvesting systems of *Prochlorococcus* are significantly different from other cyanobacteria (Wiethaus et al. 2010). Therefore, a comprehensive identification of the protein complexes in photosynthetic cells is critical for understanding the regulation of photosynthesis and other metabolic processes.

The number of sequenced photosynthetic organisms has been growing rapidly with advances in DNA sequencing technologies. The availability of whole genomic sequences and the development of mass spectrometry-based identification techniques have enabled us comprehensive protein identification in many photosynthetic organisms. For example, the large-scale identification of protein-protein interactions has achieved significant results using a model plant *Arabidopsis thaliana*(e.g. (Arabidopsis Interactome Mapping Consortium 2011) and see also a recent review by Braun et al. (Braun et al. 2013)). In another example, for cyanobacteria, a protein-protein interaction database of a model cyanobacteria *Synechocystis* sp. PCC 6803 is also constructed (Kim et al. 2008). However, the methods for large-scale identification of protein-protein interactions are still labor-intensive and time-consuming. Therefore, a rapid, sensitive, less laborious, and comprehensive approach for identifying protein complexes is necessary to obtain a better understanding of cellular processes in growing number of sequenced photosynthetic organisms.

Blue native PAGE (BN-PAGE) is developed for the high-resolution separation method of protein complexes in their structurally and enzymatically intact forms (Schägger and von Jagow 1991). The method has been shown as an useful method to separate protein complexes, especially for large, labile, and membrane protein complexes (Wittig and Schägger 2008). The general workflow for identification of protein complexes comprises the separation of protein complexes by one-dimensional (1D) BN-PAGE followed by a denaturing second-dimensional (2D) electrophoresis by SDS-PAGE, in which each protein complex dissociate into each individual protein. Then, stained protein spots with CBB, silver, or fluorescence dyes were identified by LC-MS/MS. Alternatively, the modified method of BN-PAGE coupled with LC-MS/MS has recently been developed. Fandino et al. firstly demonstrated that the BN-PAGE directly coupled with LC-MS/MS approach can identify the membrane protein complexes (Fandino et al. 2005). Helbig et al. and Wessels et al. extended the method to cover the whole length of 1D BN-lanes for LC-MS/MS (Helbig et al. 2009; Wessels et al. 2009). They demonstrated that coupling of the method with protein migration/correlation profiling is useful for finding potentially interacting proteins using mitochondrial membranes isolated from the yeast (Helbig et al. 2009) and the human embryonic kidney 293 (Wessels et al. 2009). The application of the approach to the tobacco BY-2 cells (Remmerie et al. 2011) and rat hart mitochondria (Heide et al. 2012) has been also reported.

In the first step of the method, protein complexes are solubilized by a mild detergent, such as dodecyl maltoside, and separated by BN-PAGE. Second, the BN gel is horizontally cut into slices, and the proteins in each BN gel slice are in-gel digested with trypsin and identified by LC-MS/MS. Then, the similarities between the protein migration profiles across gel slices, which are generated based on estimations of the protein abundances using label-free semi-quantitative methods, can be used to estimate potential protein-protein interactions. Comparing to the standard 2D-BN/SDS-PAGE coupled with LC-MS/MS, the omission of the second step SDS-PAGE has some advantages. The principle improvement of the method is that the rapid and simple approach from BN-gels to LC-MS/MS allows us the analysis of protein complexes without labor-intensive and time-consuming step, which is suitable for semi high-throughput analysis. In addition, the BN-PAGE coupled with LC-MS/MS does not suffer incomplete spot detection due to the limited dynamic range of the staining method (Remmerie et al. 2011). Furthermore, artifactual methionine oxidation by 2D-SDS-PAGE is prevented (Fandino et al. 2005).

In this study, we applied the BN-PAGE coupled with LC-MS/MS method to *Arabidopsis* thylakoid membranes and the whole cells of cyanobacterium *Synechocystis* sp. PCC 6803, model organisms widely used in photosynthesis studies, to systematically identify protein complexes and to construct a comprehensive and a user-friendly database. The web database helps users obtain information on selecting protein complexes of interest and to identify unknown protein complexes in *Arabidopsis* thylakoid membranes and in cells of *Synechocystis* sp. PCC 6803.

## Results

### Identification of isolated Arabidopsis thylakoid membrane proteins

**Figure 1:**
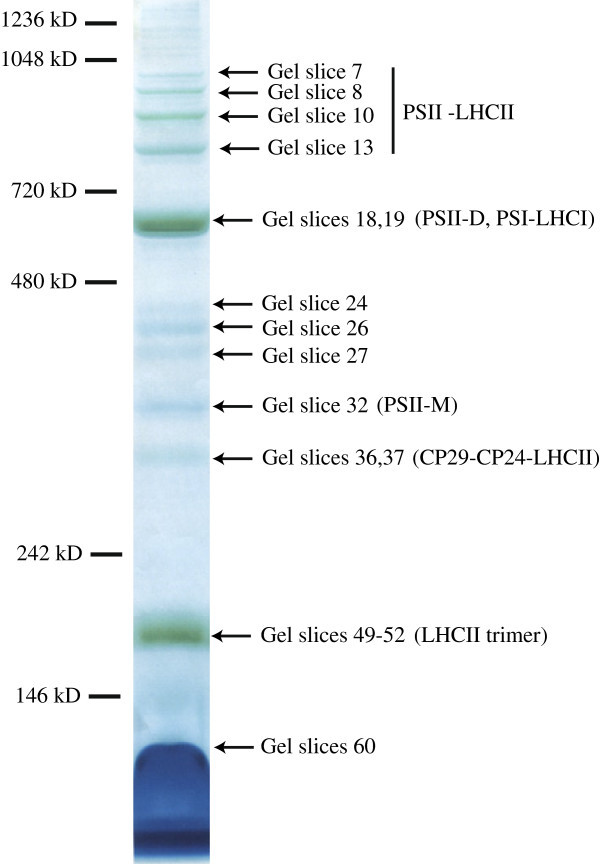
**Separation of the protein complexes in wild**-**type thylakoid membranes.** Thylakoid proteins (4 μg chlorophyll equivalent) were separated by BN-PAGE (4-13%). The BN-PAGE gel was cut horizontally into 60 pieces at approximately 1mm intervals, followed by protein identification using LC-MS/MS. The identification of the pigment protein complexes (PSII-LHCII supercomplexes (PSII-LHCII), PSI-LHCI supercomplex (PSI-LHCI), PSII dimer (PSII-D), PSI-LHCI, PSII monomer (PSII-M), CP29-CP24-LHCII trimer, and LHCII trimer) was performed according to previous reports (Takabayashi et al. 2011). The gel slices that corresponded to the pigment protein complexes are indicated.

We purified the *Arabidopsis* thylakoid membranes using sucrose density gradients. The membranes were then solubilized with dodecyl maltoside and separated by BN-PAGE. The band pattern of the separated thylakoid protein complexes in the CBB staining gel was essentially the same as the profile of WT identified in our previous report (Takabayashi et al. 2011). Based on the identifications in that report, the bands of the four PSII-LHCII supercomplexes, the dimeric PSII and the PSI-LHCI supercomplex, the PSII monomer, the LHCII assembly (the CP29-CP24-LHCII trimer), and the trimeric LHCII visualized by CBB staining were identified (Figure 1).

To identify the thylakoid proteins separated by BN-PAGE, we sliced a BN gel lane horizontally into 60 pieces from the top of the gel to immediately before the dye front (Figure 1). These gel slices were processed for in-gel digestion with trypsin and analyzed by LC-MS/MS. In total, 245 proteins were identified from the 60 gel slices (Additional file 1: Table S1). The purified thylakoid membranes may have been contaminated by other cellular fractions. To estimate the number of thylakoid-localized proteins among all the identified proteins, we compared our results with the recent large-scale thylakoid proteomic study by Ferro et al. (Ferro et al. 2010). According to their results, 102 of the 245 proteins were classified as thylakoid proteins, and 33 proteins were classified as envelope or stromal proteins (Additional file 1: Table S1). In addition, we identified 15 known thylakoid-localized proteins that were not classified as thylakoid proteins by Ferro et al. (Ferro et al. 2010), including NDH proteins (At1g14150, At1g70760, At2g39470, At5g43750, At5g58260, AtCg-00420, AtCg00430, AtCg01010, AtCg01090), Lil3 proteins (At4g17600, At5g47110), Lhca5 (At1g45474), LPA3 (At1g73060), STN7 (At1g68830), and YCF4 (AtCg00520). Therefore, a minimum of 117 of the 245 proteins (48%) were likely thylakoid-localized proteins, whereas the functions of the remaining 95 proteins were largely unknown.

To verify the enrichment of the thylakoid proteins in our protein sample, we estimated the abundances of the identified proteins in the protein samples using the emPAI (exponentially modified protein abundance index) method (Ishihama et al. 2005). The emPAI method, which is based on the numbers of observed peptides per protein by LC-MS/MS and is further normalized by the observable number of peptides, has been used for label-free semi-quantitative estimates of relative protein abundances and has been reported to exhibit a good correlation with protein abundance in samples (Ishihama et al. 2005). According to the paper, we calculated the protein content in molar percentages using the emPAI values (Additional file 1: Table S1) and found that the 117 putative thylakoid proteins in our results accounted for 97% of all the identified proteins, suggesting that the thylakoid proteins were substantially enriched in the sample.

### The emPAI-based profiles of the subunits in the thylakoid large protein complexes were well correlated with their separation patterns in BN-PAGE

The protein migration profiles, which were assessed by the label-free estimation of protein abundances versus the migration distance in BN-PAGE, were reported to be substantially similar among subunits in the same protein complexes (Helbig et al. 2009; Wessels et al. 2009; Remmerie et al. 2011). Label-free quantitative approaches are inexpensive and have a considerable analytical depth and dynamic range, although quantitative precision is typically lower compared to stable-isotope approaches (Schulze and Usadel 2010).

In this study, we generated the emPAI-based protein migration profiles across all the BN gel slices, in which each emPAI value was plotted on the y-axis, and each gel slice number (ordered from top to bottom) was plotted on the x-axis, based on the Mascot data from each gel slice (Additional file 2: Table S2). Before using the emPAI-based protein migration profiles for identifying protein complexes, we first verified the correlation between the emPAI-based protein migration profiles and their band patterns by comparing the emPAI-based migration profiles of the PSII proteins with their band patterns, which were visualized by the CBB staining on the BN-PAGE.

**Figure 2:**
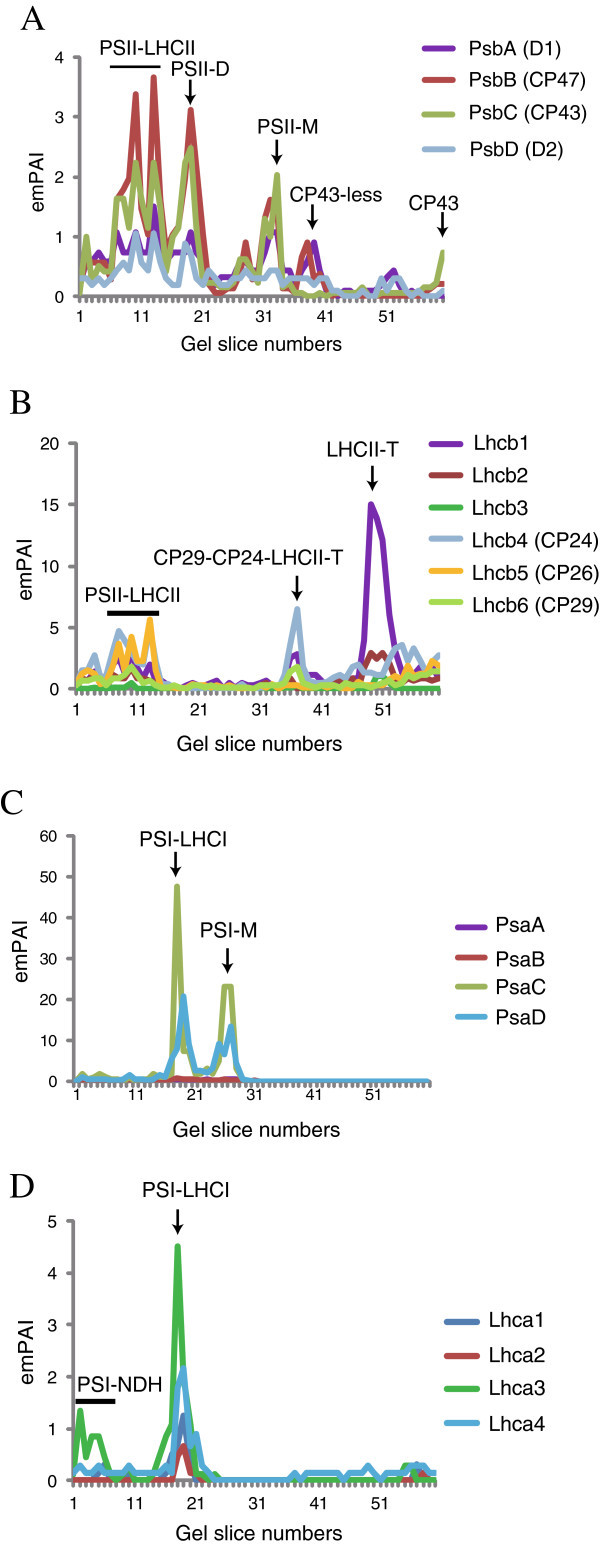
**Protein migration profiles of the PSII, LHCII, PSI, and LHCI proteins.** The emPAI-based protein migration profiles of the PSII core subunits (PsbA, PsbB, PsbC, and PsbD) (**A**), LHCII proteins (Lhcb1-6) (**B**), four PSI core subunits (PsaA, PsaB, PsaC, and PsaD) (**C**), and LHCI proteins (Lhca1-4) (**D**) are shown. The emPAI value of PsaD was the sum of the emPAI values of PsaD1 and PsaD2. The pigment protein complexes corresponding to the peaks in the migration profiles of the PSI-NDH supercomplex (PSI-NDH), PSII-LHCII supercomplex (PSII-LHCII), PSI-LHCI supercomplex (PSI-LHCI), PSII dimer (PSII-D), PSI-LHCI, PSII monomer (PSII-M), CP29-CP24-LHCII trimer, and LHCII trimer) are indicated.

The bands of the PSII-LHCII supercomplexes, the PSII dimer, and the PSII monomer were visualized by the CBB staining in the region on the BN-PAGE gel lane corresponding to the slices between 7 and 13, slices 18 and 19, and slice 32, respectively (Figure 1). Meanwhile, the emPAI-based migration profiles of four PSII core subunits (D1 (PsbA), CP47 (PsbB), CP43 (PsbC), and D2 (PsbD)) had peaks that corresponded to the bands of the PSII complexes from the BN-PAGE (Figure 2A). The emPAI-based migration profiles of the remaining PSII core proteins (PsbE, PsbH, and PsbL) were also similar (Additional file 3: Figure S1). Likewise, the emPAI-based migration profiles of LHCII proteins had peaks that corresponded to the PSII-LHCII supercomplex (the slices between 7 and 13), the CP29-CP24-LHCII supercomplex (slices 36 and 37), and the LHCII trimer (the slices between 49 and 52) in the BN-PAGE, respectively (Figure 2B). The emPAI values in the peaks of PsbA and PsbD were much smaller than those of PsbC and PsbC (Figure 2A), likely because their hydrophobicity. The likelihood of the MS-based protein detection varied greatly depending on the properties of each peptide, such as the peptide length, net charge, and solubility (Lu et al. 2007). Especially, the probability of identifying very hydrophobic proteins is typically low.

In addition to PSII, the PSI-LHCI supercomplex (the slices between 17 and 20) and the PSI monomer (the slices between 26 and 28) were visualized in BN-PAGE by the CBB staining (Figure 1), and the emPAI-based migration profiles of four PSI core proteins (PsaA, PsaB, PsaC, and PsaD) had peaks that corresponded to the CBB-stained bands of the PSI-LHCI supercomplex and the PSI monomer (Figure 2C). The emPAI values of PsaA/PsaB were much smaller than those of PsaC and PsaD, likely because their hydrophobicity. All of the remaining PSI proteins, except for PsaN, exhibited similar emPAI-based migration profiles (Additional file 3: Figure S1). The emPAI-based migration profiles of the LHCI proteins also contained major peaks corresponding to the PSI-LHCI supercomplex (Figure 2D).

Although the large thylakoid protein complexes, cytochrome *b*_*6*_/*f* complex, ATP synthase, and the NDH complex, were not clearly visualized in the BN gel (Figure 1), we previously reported their oligomeric states in the BN gel using 2D-BN/SDS-PAGE (Takabayashi et al. 2011). Consequently, we further verified whether the emPAI-based migration profiles of the subunits in the large thylakoid protein complexes were consistent with their respective oligomeric states.

**Figure 3:**
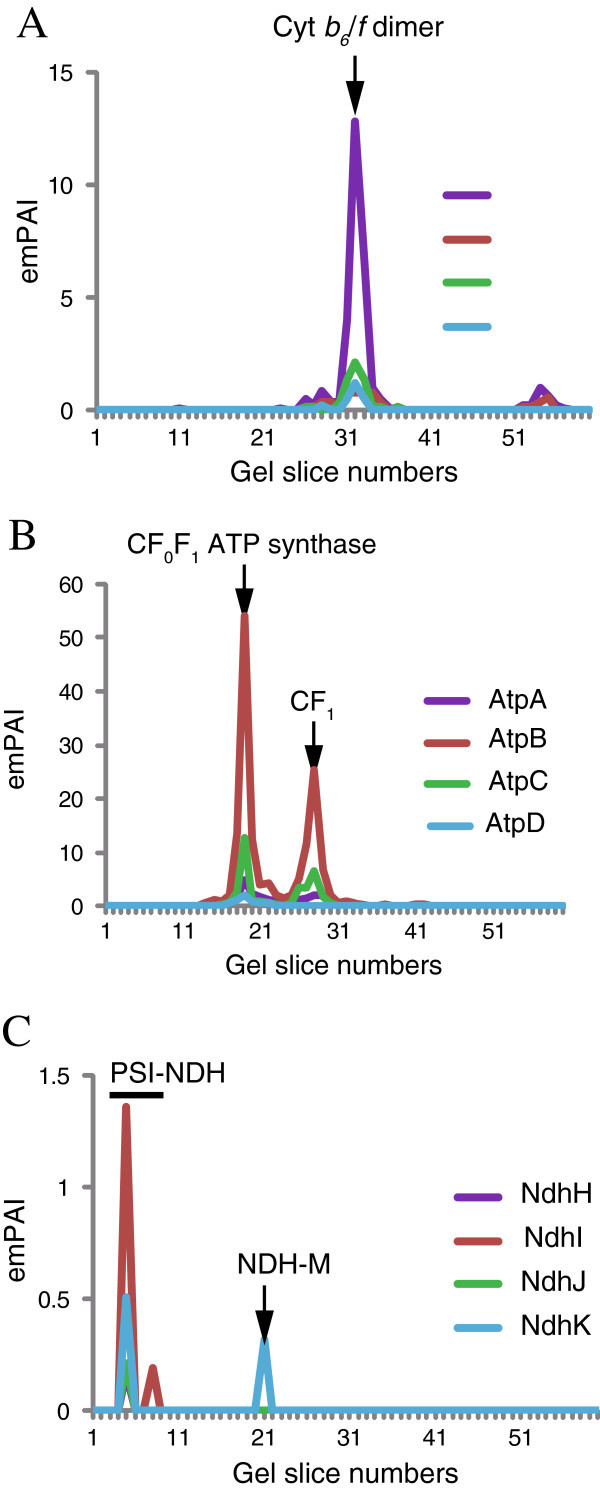
**Protein migration profiles of four representative subunits from the cytochrome*b***_***6***_***/f*, ATP synthase, and NDH proteins.** The emPAI-based protein migration profiles of the cytochrome *b*_*6*_/*f* proteins (PetA, PetB, PetC, and PetD) (**A**), ATP synthase proteins (AtpA, AtpB, AtpC, and AtpD) (**B**), and NDH proteins (NdhH, NdhI, NdhJ, and NdhK) (**C**) are shown. The protein complexes corresponding to the peaks in the migration profiles of the cytochrome *b*_*6*_/*f* dimer, CFoF_1_ATP synthase, CF_1_ subcomplex, PSI-NDH supercomplex (PSI-NDH), and NDH monomer (NDH) were estimated based on the oligomeric states of the protein complexes. For the NDH proteins, we followed the nomenclature proposed by Ifuku et al. (2011) (Ifuku et al. 2011).

The emPAI-based migration profiles of the four proteins of cytochrome *b*_*6*_/*f* complex (PetA, PetB, PetC, and PetD) contained a peak that likely corresponded to the dimeric form of the complex at gel slice 32 (Figure 3A), and the emPAI-based migration profiles of the chloroplastic ATP synthase proteins (AtpA, AtpB, AtpC1, AtpD, AtpE, AtpF, AtpI, and AT4G32260) had a peak that corresponded to the CF_0_F_1_ complex at gel slice 19 and some of them (AtpA, AtpB, AtpC1, and AtpE) also had a peak that corresponded to the CF_1_ subunit at gel slice 28 (Figure 3B and Additional file 3: Figure S1).

In addition to the major protein complexes of the thylakoid membranes, 15 NDH proteins were identified in this study (Additional file 1: Table S1). The nomenclature of NDH proteins were followed by Ifuku et al. (2011). The major form of the NDH complex in *Arabidopsis* is the PSI-LHCI supercomplex, which consists of the PSI-LHCI and the monomeric NDH, with Lhca5 and Lhca6 proteins acting as linker proteins (Ifuku et al. 2011). The emPAI-based migration profiles of all the identified NDH subunits, except PnsL5, contained a peak in slice 5 (above the bands of the PSII supercomplexes) (Figure 3C and Additional file 4: Figure S2) that corresponded to the PSI-NDH supercomplex. Many of the PSI and LHCI proteins were also identified in slice 5. In addition, PnsB1, PnsB2, PnsB5, and PnsL3 proteins were also identified in the slices between 11 and 13 (Figure 3C and Additional file 4: Figure S2), likely corresponding to the monomeric NDH complex.

These results demonstrated that the subunits from the same protein complex generally exhibited similar emPAI-based migration profiles. In addition, the emPAI-based protein migration profiles can be used to reveal the oligomeric states of the photosynthetic protein complexes and used to find the co-migrated proteins with a protein of interest.

However, it should be noted that some of the PSI and PSII subunits were not identified in this study. Since the probability of identifying a small protein and a very insoluble protein were typically low, the small subunits of the PSI and the PSII may be difficult to identify by LC-MS/MS. Future technical developments should be required to comprehensive identify protein complexes.

### Analysis of the whole cell proteins of *Synechocystis sp*. *PCC 6803*

Using *Arabidopsis* thylakoid membranes as a model, our data presented here showed that BN-PAGE coupled with LC-MS/MS was useful for identifying potential protein complexes. One of the goals of our research is the comprehensive identification of protein complexes in the whole cells of photosynthetic organisms to aid in understanding their cellular processes. Therefore, we applied the described method to the whole cell proteins of the cyanobacterium *Synechocystis* sp. PCC 6803.

**Figure 4:**
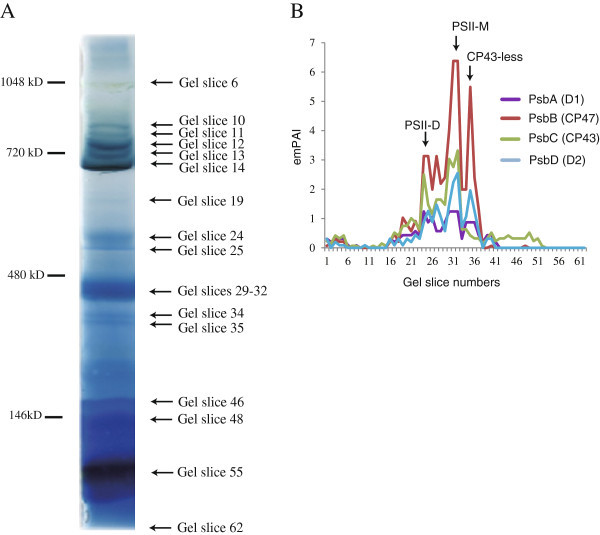
**The application of the BN-PAGE method coupled with LC-MS/MS to*Synechocystis cells.*** Separation of the protein complexes from *Synechocystis* cells by BN-PAGE. Protein bands were visualized by CBB-staining (**A**). Protein migration profiles of PSII core subunits in *Synechocystis* (**B**). Putative peaks corresponding to the PSII dimer (PSII-D), PSII monomer (PSII-M), and CP43-less PSII were shown.

The whole cells of *Synechocystis* sp. PCC 6803 were homogenized using glass beads on a vortex mixer. DNase treatment was performed to remove the genomic DNA that was reported to significantly interfere with BN-PAGE using the whole cells of bacteria (Liang et al. 2009). The protein complexes were mildly solubilized with dodecyl maltoside and then separated by BN-PAGE (Figure 4). The BN gel lanes were cut horizontally from the top of the gel to just before the dye front, and the gel slices were then processed by in-gel digestion with trypsin and analyzed by LC-MS/MS. As a result, we identified 1,458 *Synechocystis* proteins, which were accounting for 41% of the total number of all the predicted gene models (Additional file 5: Table S3). According to the functional category by CyanoBase (http://genome.kazusa.or.jp/cyanobase), identified proteins were distributed all over functional categories (Additional file 6: Table S4). Notably, 554 proteins were classified into “Hypothetical” or “Unknown” (Additional file 6: Table S4).

We then generated the emPAI-based migration profiles for identified proteins based on the Mascot data from each gel (Additional file 7: Table S5). For examples, the peaks in their migration profiles corresponding to the putative PSII dimer (slices 24 and 25), the PSII monomer (slices 31 and 32), and the CP43-less PSII (the slice 35) were well correlated with the oligomeric states of PSII in *Synechocystis* according to the previous paper (Herranen et al. 2004) (Figure 4B). These data shows that the BN-PAGE coupled with LC-MS/MS was useful, even when whole cells were used.

### Building a Protein Co-migration Database for Photosynthetic Organisms (PCoM-DB)

**Figure 5:**
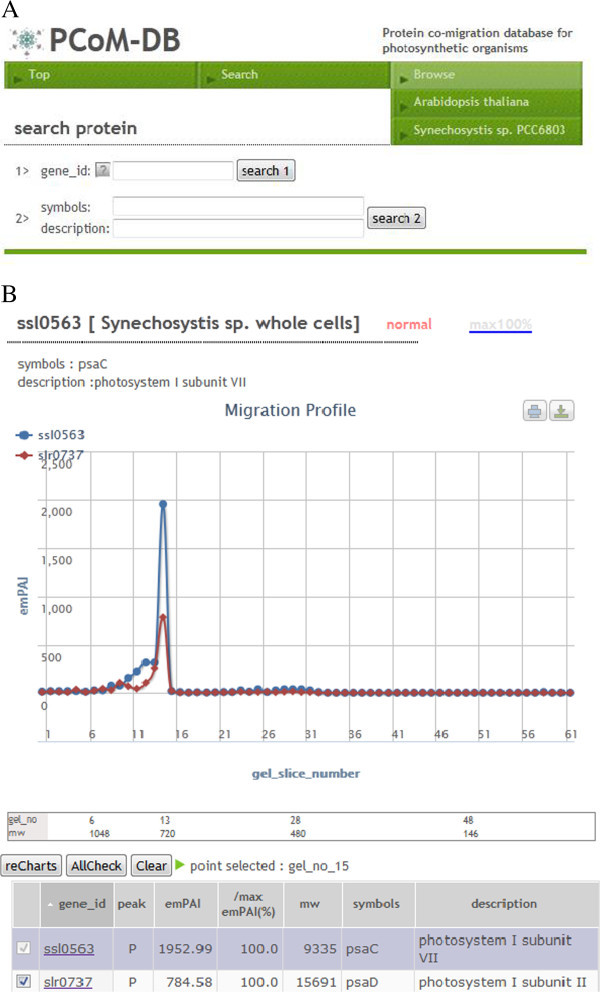
**Browse and search interfaces in PCoM-DB (A), and comparing the migration profiles of*Synechocystis*PsaC and PsaD proteins using PCoM-DB (B).** Users can easily find the protein migration profile of a protein of interest through browse and search interfaces (**A**). Then, users also can find the potential interacting partners by finding the proteins which shared the peaks in the protein migration profiles using PCoM-DB (**A**). In addition, users can compare the overall protein migration profiles of potential interacting partners with a protein of interest. In this case, protein migration profiles of PsaC and PsaD were shown as an example (**B**). The migration profiles of a *Synechocystis* PsaC protein had a peak in the gel slice 15, corresponding to the PSI trimer. Only clicking the blue closed circle showing the PsaC emPAI value at the gel slice 15, users can find the proteins which were also detected in the gel slice 15 as potential interacting partners (and one of them was PsaD).

Because emPAI-based migration profiles provide us useful information regarding protein complexes, we built a web application called PCoM-DB (http://pcomdb.lowtem.hokudai.ac.jp/proteins/top) that shows and compares the migration profiles of the identified proteins in this study (Figure 5).

PCoM-DB is freely accessible through the web browsers. The emPAI-based migration profiles shown in PCoM-DB provide users the following information regarding identified proteins: 1) the number of putative protein complexes associated with a protein of interest; 2) the estimated molecular sizes of the protein complexes associated with the protein of interest; and 3) the relative accumulation levels of each protein complex associated with the protein of interest. In addition, users can find co-migrated proteins with a protein of interest, based on the assumption that the proteins whose peaks in their migration profile overlap within the same gel slice possibly interact with each other. PCoM-DB helps users to find the potential interacting partners with a protein of interest.

## Discussion

In this study, we analyzed thylakoid and cyanobacterial protein complexes using the BN-PAGE coupled with LC-MS/MS. We found that the protein migration profiles of the PSII, PSI, cytochrome *b*_*6*_/*f*, ATP synthase, and NDH subunits in *Arabidopsis* thylakoids were well correlated with their oligomeric states within the BN gel. Also, the protein migration profiles of the PSII core proteins in *Synechocystis* cells were similar with each other, and should be correlated with the oligomeric states of PSII in the previous report.

BN-PAGE coupled with LC-MS/MS is expected to be much more sensitive than 2D-BN/SDS-PAGE followed by MS analysis because the sensitivities of the CBB, silver, and fluorescent staining used for the visualization of protein spots are lower than the sensitivity of protein identification with LC-MS/MS. However, the likelihood of the MS-based protein detection varied greatly depending on the properties of each peptide. Especially, LC-MS/MS typically has difficulty in detecting very hydrophobic proteins. Therefore, thylakoid membrane protein complexes may not be suitable for comprehensive identification by LC-MS/MS. On the other hand, 1,458 proteins were identified in the whole cells of *Synechocystis*. This number is equivalent to 41% of all predicted gene model, even though some proteins may not always expressed in *Synechocystis* cells. According to the functional categories in CyanoBase, identified proteins in *Synechocystis* cells distributed all over functional categories and well contained the unknown proteins. We think that the low number of total proteins encoded in bacterial genome has a great advantage for high sensitivity in LC-MS/MS, because the protein identification probability by searching sequence databases using mass spectrometry data should be higher. Our data presented here showed that BN-PAGE coupled with LC-MS/MS method is expected to be suitable for the large-scale identification of protein complexes, especially in bacterial cells.

Finally, we built a web database called PCoM-DB to make our data publicly accessible online. PCoM-DB shows users the migration profiles of proteins of interest. The protein migration profiles provide important information regarding the protein complexes. First, the protein migration profiles display the number and the estimated sizes of the protein complexes associated with a protein of interest. Second, they also provide a list of the co-migrated proteins whose protein migration profile peaks were overlapped with those of the protein of interest, or whose migration profiles were similar to those of the protein of interest. The co-migrating proteins with a protein of interest are potential interacting partners with the protein of interest. Different sources of biological information, such as gene expression data, protein domain information data, and mutant phenotypes, can be used for the further selection of the potential interacting partners.

At present, our database contains the data presented here (*Arabidopsis* thylakoid membrane proteins and *Synechocystis* sp. PCC 6803 proteins). We plan to add data to PCoM-DB from other cyanobacteria in addition to additional compartments from *Arabidopsis*. The accumulation of the data in PCoM-DB should help researchers to find unknown protein complexes.

## Methods

### Growth conditions

The Columbia ecotype of *Arabidopsis thaliana* was used as the wild type in this study. The wild-type plants were grown in soil under constant illumination (70 μmol photon m^-2^ s^-1^) at 23°C. The growth conditions of *Synechocystis* sp. PCC 6803 were described previously (Satoh et al. 2001).

### Isolation of *Arabidopsis* thylakoid membranes

All of the following procedures were conducted at 4°C unless otherwise stated. The thylakoid membranes were isolated from the leaves of 4-week-old plants. The thylakoid membranes were prepared essentially according to the methods described by Salvi et al. (Salvi et al. 2008). Briefly, the leaves were homogenized in isolation buffer containing 20 mM Tricine-NaOH (pH 8.0), 0.45 M sorbitol, 10 mM EDTA-2Na, 1 mM NaHCO_3_, 0.1% (w/v) BSA, 0.05% (w/v) DTT, and 0.05% (w/v) PVP. The homogenate was filtered through four layers of Miracloth and centrifuged at 1,000 ×*g* for 5 min. The pellet was suspended in wash buffer containing 20 mM MOPS-NaOH (pH 7.6), 0.33 M sorbitol, 5 mM MgCl_2_, and 2.5 mM EDTA-2Na. After a Percoll density gradient centrifugation, the intact chloroplasts were collected from the interface between 40% and 80% Percoll. The intact chloroplast suspension was washed twice and then osmotically ruptured in swelling buffer containing 10 mM MOPS-NaOH (pH 7.6) and 4 mM MgCl_2_. After a sucrose density gradient (2 M, 0.93 M and 0.6 M) centrifugation at 95,000 ×*g* in a RPS56T rotor (Hitachi, Tokyo, Japan) for 60 min at 4°C, the purified thylakoid membranes were collected from the interface between 0.93 M and 2 M sucrose.

### BN-PAGE

BN-PAGE was performed essentially according to the methods described by Wittig et al. (Wittig et al. 2006). The chlorophyll content was determined according to Porra et al. (Porra et al. 1989).

Thylakoid membranes purified from *Arabidopsis* were suspended at a concentration of 1 mg/ml chlorophyll *a* and *b* in ice-cold resuspension buffer containing 50 mM imidazole-HCl (pH 7.0), 20% glycerol, 5 mM 6-aminocaproic acid, and 1 mM EDTA-2Na. Cells of *Synechocystis* were harvested by centrifugation and homogenized using 0.1 mm glass beads in a vortex mixer in ice-cold resuspension buffer. The genomic DNA was removed using Benzonase Nuclease (Merck, Darmstadt, Germany) for 30 min on ice. The suspension (1 mg/ml chlorophyll) was solubilized with 1% (w/v) n-dodecyl-β-D-maltoside on ice for 5 min. After centrifugation at 18,800 ×*g* for 10 min at 4°C, the supernatants were supplemented with Coomassie Blue solution [5% (w/v) Serva Blue G (Serva, Heidelberg, Germany), 500 mM 6-aminocaproic acid, and 50 mM Imidazole-HCl (pH 7.0)] to a detergent/Coomassie ratio of 4/1 (w/w). The samples (4 μg chlorophyll equivalent) were separated in 4-13% polyacrylamide gradient gels at 4°C for 14 h at 40 V.

### Nano-LC/MS/MS for proteomics of *Arabidopsis* thylakoids and *Synechocystis* cells

The BN-PAGE gel lanes were cut horizontally into approximately 60 slices at regular intervals (ca. 1 mm) from the top of the gel to just before the dye front. All gel slices were treated by in-gel digestion with trypsin (Roche) according to Shevchenko et al. (Shevchenko et al. 1996).

LC-MS/MS analyses were performed using an LTQ ion-trap MS (Thermo Fisher Scientific, Yokohama, Japan) coupled with an HPLC (AMR Inc., Tokyo, Japan) and a nano-spray electrospray ionization device (Michrom Bioresources Inc., CA, USA), essentially according to Kasahara et al. (2012) (Kasahara et al. 2012). The resulting peptides were loaded onto an L-column 2 ODS column packed with C18 (5 μm, 12 nm pore size) (Chemicals Evaluation and Research Institute, Japan) and separated by a gradient using solvent A (2% acetonitrile in 0.1% formic acid) and solvent B (90% acetonitrile in 0.1% formic acid). The gradient condition was 5% B to 90% B and the flow rate was 1 μL/min. The separated peptides were analyzed with an LTQ for MS/MS analysis. The ESI conditions for the LTQ instrument were set as follows: capillary temperature of 200°C and a spray voltage of 2.0 kV. The scan range was m/z 450–1800 for LTQ. The scan time for MS and MS/MS were 0.6 s. The ion selection threshold, the minimum signal required for MS/MS, was set to 200 counts. MS/MS spectra were acquired in data-dependent scan mode. After the full spectrum scan, one MS/MS spectrum of the single most intense peaks was also collected.

Protein identification was performed as follows. Raw files were converted to a Mascot generic file (mgf) and searched with Mascot version 2.2 (Matrix Science) into the TAIR9 database (The *Arabidopsis* Information Resource; http://www.arabidopsis.org; Total sequences, 33410; Total residues, 13434913) for *Arabidopsis* thylakoid proteins or searched with the Mascot version 2.3 into the protein data from CyanoBase (http://genome.kazusa.or.jp/cyanobase/; Total sequences, 3569; Total residues, 1137101) for *Synechocystis* proteins. The results were filtered with a significance threshold of p < 0.05 for peptides identifications and the MudPIT scoring algorism was used. Also, the following search parameters were used: 0.05 for ions score cut off, 1.2 Da peptide tolerance; ±0.8 Da MS/MS tolerance; 1+, 2+, or 3+ peptide charge; trypsin digestion with two missed cleavage allowed; carbamidomethyl modification of cysteines as a fixed modification; and oxidation of methionine as a variable modification. Furthermore, only the top scoring peptide match was used in this study and we accepted proteins identified by at least one unique peptide. False discovery rates (FDR) for peptide matches above the identity threshold were 4.54% for *Arabidopsis* thylakoids and 2.83% for *Synechocystis* cells, respectively. The detailed Mascot data for identified peptides were shown in Additional file 8: Table S6 for *Arabidopsis* thylakoids and Additional file 9: Table S7 for *Synechocystis* cells.

## Conclusion

Here, we applied the BN-PAGE followed by LC-MS/MS method for comprehensive identification of protein complexes into the thylakoid protein complexes of *Arabidopsis* and whole cell protein complexes of *Synechocystis* sp. PCC 6803. The emPAI-based protein migration profiles based on the data from the method were useful for finding protein complexes, especially for *Synechocystis*. The web database called PCoM-DB to make our data publicly accessible online has been constructed. PCoM-DB, which stores the analyzed data with a user-friendly interface, can help users to find novel protein complexes in *Arabidopsis* thylakoids and *Synechocystis*.

## Electronic supplementary material

Additional file 1: Table S1: Identified proteins from *Arabidopsis* thylakoids. (DOC 360 KB)

Additional file 2: Table S2: The emPAI values across BN gel slices of the identified proteins from *Arabidopsis* thylakoids. (XLS 150 KB)

Additional file 3: Figure S1: Protein migration profiles of the remaining subunits of the PSII, PSI, and ATP synthase proteins. The emPAI-based protein migration profiles of the remaining PSII core subunits (PsbE, PsbH, and PsbL) (A), the remaining PSI proteins (PsaE, PsaF, PsaG,PsaH2, PsaK, PsaL, and PsaN) (B), and the remaining ATP synthase subunits (AtpE, AtpF, AtpI, and AT4G32260) (C). (PDF 452 KB)

Additional file 4: Figure S2: Protein migration profiles of the remaining subunits of the NDH proteins. The emPAI-based protein migration profiles of the six NDH proteins (NdhE, NdhF, NdhL, NdhN, NdhU, and PnsB1) (A) and the remaining NDH proteins (PnsB2, PnsB5, PnsL5, PnsL1, PnsL2, and PnsL3) (B). (PDF 364 KB)

Additional file 5: Table S3: Identified proteins from *Synechocystis* cells. (DOC 1 MB)

Additional file 6: Table S4: The emPAI values across BN gel slices of the identified proteins from *Synechocystis* cells. (XLS 28 KB)

Additional file 7: Table S5: Functional classification of identified *Synechocystis* proteins according to CyanoBase. (XLS 734 KB)

Additional file 8: Table S6: Mascot data for identified peptides from *Arabidopsis* thylakoids. (XLSX 1 MB)

Additional file 9: Table S7: Mascot data for identified peptides from *Synechocystis* cells. (XLSX 9 MB)
