# Supplementary material for: Protein co-migration database (PCoM -DB) for Arabidopsis thylakoids and Synechocystis cells
Source: Springerplus. 2013 Apr 8;2:148. doi: 10.1186/2193-1801-2-148 (PMC3647082; doi:10.1186/2193-1801-2-148)
Supplement: Supplementary file 1 — Additional file 1: Table S1: Identified proteins from Arabidopsis thylakoids. (DOC 360 KB) [file 40064_2013_228_MOESM1_ESM.doc]

**Additional File 1: Table S1.** Identified proteins from *Arabidopsis* thylakoids

| Locus | Symbols | TAIR9 Description | AT_CHLORO  Localization | emPAI | PC (mol%) |
| --- | --- | --- | --- | --- | --- |
| AT1G03130 | PSAD2 | photosystem I subunit D-2 | THY-ENV | 39.8 | 2.99 |
| AT1G03630 | PORC | protochlorophyllide oxidoreductase C | ENV-THY | 0.58 | 0.04 |
| AT1G05790 |  | lipase class 3 family protein |  | 0.4 | 0.03 |
| AT1G06430 | FTSH8 | FTSH protease 8 | THY | 0.45 | 0.03 |
| AT1G06680 | PSBP1 | photosystem II subunit P-1 | THY | 0.13 | 0.01 |
| AT1G06950 | TIC110 | translocon at the inner envelope membrane of chloroplasts 110 | ENV | 0.36 | 0.03 |
| AT1G07660 |  | Histone superfamily protein | THY-STR | 0.35 | 0.03 |
| AT1G12900 | GAPA2 | glyceraldehyde 3-phosphate dehydrogenase A subunit 2 | STR-ENV | 0.82 | 0.06 |
| AT1G14150 | PNSL2 | PsbQ-like 2 |  | 0.17 | 0.01 |
| AT1G14345 |  | NAD(P)-linked oxidoreductase superfamily protein | THY | 0.18 | 0.01 |
| AT1G15820 | LHCB6 | light harvesting complex photosystem II subunit 6 | THY | 31.59 | 2.37 |
| AT1G15980 | PNSB1 | NDH-dependent cyclic electron flow 1 | THY | 1.28 | 0.1 |
| AT1G16030 | Hsp70b | heat shock protein 70B |  | 0.05 | 0 |
| AT1G16590 | REV7 | DNA-binding HORMA family protein |  | 0.16 | 0.01 |
| AT1G18170 |  | FKBP-like peptidyl-prolyl cis-trans isomerase family protein |  | 0.28 | 0.02 |
| AT1G20020 | FNR2 | ferredoxin-NADP(+)-oxidoreductase 2 | STR-THY | 2.47 | 0.19 |
| AT1G20400 |  | Protein of unknown function (DUF1204) |  | 0.03 | 0 |
| AT1G29910 | LHCB1.2 | chlorophyll A/B binding protein 3 | THY | 39.92 | 3 |
| AT1G29930 | LHCB1.3 | chlorophyll A/B binding protein 1 | THY | 20.57 | 1.54 |
| AT1G30380 | PSAK | photosystem I subunit K | THY | 1.2 | 0.09 |
| AT1G31330 | PSAF | photosystem I subunit F | THY-ENV | 27.22 | 2.04 |
| AT1G32080 |  | membrane protein, putative | ENV | 0.07 | 0.01 |
| AT1G32750 | HAF13 | HAC13 protein (HAC13) |  | 0.04 | 0 |
| AT1G34000 | OHP2 | one-helix protein 2 | THY | 0.2 | 0.02 |
| AT1G35680 | RPL21 | Ribosomal protein L21 | STR-ENV | 0.34 | 0.03 |
| AT1G43980 |  | Tetratricopeptide repeat (TPR)-like superfamily protein |  | 0.3 | 0.02 |
| AT1G44575 | PSBS | Chlorophyll A-B binding family protein | THY | 17.98 | 1.35 |
| AT1G45474 | LHCA5 | photosystem I light harvesting complex gene 5 |  | 0.9 | 0.07 |
| AT1G50250 | FTSH1 | FTSH protease 1 | THY-ENV | 0.83 | 0.06 |
| AT1G51110 |  | Plastid-lipid associated protein PAP / fibrillin family protein | THY-ENV | 0.24 | 0.02 |
| AT1G52230 | PSAH2 | photosystem I subunit H2 | THY | 6.99 | 0.53 |
| AT1G52450 |  | Ubiquitin carboxyl-terminal hydrolase-related protein |  | 0.03 | 0 |
| AT1G52510 |  | alpha/beta-Hydrolases superfamily protein | STR-ENV | 0.09 | 0.01 |
| AT1G54350 |  | ABC transporter family protein |  | 0.05 | 0 |
| AT1G54360 | TAF6B1 | TBP-ASSOCIATED FACTOR 6B |  | 0.07 | 0.01 |
| AT1G54780 | TLP18.3 | thylakoid lumen 18.3 kDa protein | THY | 1.51 | 0.11 |
| AT1G55040 |  | zinc finger (Ran-binding) family protein |  | 0.08 | 0.01 |
| AT1G55250 | HUB2 | histone mono-ubiquitination 2 |  | 0.04 | 0 |
| AT1G55670 | PSAG | photosystem I subunit G | THY | 15.95 | 1.2 |
| AT1G55780 |  | metal ion binding |  | 0.22 | 0.02 |
| AT1G56120 |  | Leucine-rich repeat transmembrane protein kinase |  | 0.03 | 0 |
| AT1G56500 |  | haloacid dehalogenase-like hydrolase family protein | THY-STR | 0.32 | 0.02 |
| AT1G58280 |  | Phosphoglycerate mutase family protein |  | 0.1 | 0.01 |
| AT1G58766 |  | unknown protein |  | 0.05 | 0 |
| AT1G60190 |  | ARM repeat superfamily protein |  | 0.05 | 0 |
| AT1G60490 | ATVPS34 | vacuolar protein sorting 34 |  | 0.04 | 0 |
| AT1G60850 | ATRPAC42 | DNA-directed RNA polymerase family protein |  | 0.09 | 0.01 |
| AT1G61520 | LHCA3 | photosystem I light harvesting complex gene 3 | THY | 15.61 | 1.17 |
| AT1G64770 | PNSB2 | NDH-dependent cyclic electron flow 1 | THY | 0.65 | 0.05 |
| AT1G65260 | PTAC4 | plastid transcriptionally active 4 | ENV | 0.54 | 0.04 |
| AT1G66460 |  | Protein kinase superfamily protein |  | 0.07 | 0.01 |
| AT1G67090 | RBCS1A | ribulose bisphosphate carboxylase small chain 1A | STR-ENV | 0.19 | 0.01 |
| AT1G68830 | STN7 | STT7 homolog STN7 |  | 0.3 | 0.02 |
| AT1G70760 | NDHL | inorganic carbon transport protein-related |  | 0.17 | 0.01 |
| AT1G71500 |  | Rieske (2Fe-2S) domain-containing protein | THY | 0.61 | 0.05 |
| AT1G73060 | LPA3 | Low PSII Accumulation 3 |  | 0.2 | 0.02 |
| AT1G73990 | SPPA | signal peptide peptidase | ENV-THY | 0.1 | 0.01 |
| AT1G74470 | GGR | Pyridine nucleotide-disulphide oxidoreductase family protein | THY | 5.88 | 0.44 |
| AT1G76110 |  | HMG (high mobility group) box protein with ARID/BRIGHT DNA-binding domain |  | 0.1 | 0.01 |
| AT1G77490 | TAPX | thylakoidal ascorbate peroxidase | THY | 0.08 | 0.01 |
| AT1G78630 | emb1473 | Ribosomal protein L13 family protein | STR-ENV | 0.42 | 0.03 |
| AT2G01140 |  | Aldolase superfamily protein | STR-THY | 0.29 | 0.02 |
| AT2G04750 |  | Actin binding Calponin homology (CH) domain-containing protein |  | 0.05 | 0 |
| AT2G05070 | LHCB2.2 | photosystem II light harvesting complex gene 2.2 | THY | 34.62 | 2.6 |
| AT2G05310 |  | unknown protein |  | 0.65 | 0.05 |
| AT2G05620 | PGR5 | proton gradient regulation 5 | THY | 4.32 | 0.32 |
| AT2G13640 | GONST1 | Transcription factor IIS family protein |  | 0.08 | 0.01 |
| AT2G20190 | CLASP | CLIP-associated protein |  | 0.02 | 0 |
| AT2G20260 | PSAE2 | photosystem I subunit E-2 | THY-ENV | 17.64 | 1.32 |
| AT2G21330 | FBA1 | fructose-bisphosphate aldolase 1 | STR-THY | 6.34 | 0.48 |
| AT2G22360 |  | DNAJ heat shock family protein | ENV | 0.08 | 0.01 |
| AT2G24420 |  | DNA repair ATPase-related |  | 0.35 | 0.03 |
| AT2G24820 | TIC55 | translocon at the inner envelope membrane of chloroplasts 55-II | ENV | 0.4 | 0.03 |
| AT2G27285 |  | Coiled-coil domain-containing protein 55 (DUF2040) |  | 0.1 | 0.01 |
| AT2G28900 | OEP16 | outer plastid envelope protein 16-1 | ENV | 1.06 | 0.08 |
| AT2G29140 | APUM3 | pumilio 3 |  | 0.06 | 0 |
| AT2G30950 | VAR2 | FtsH extracellular protease family | THY | 1.54 | 0.12 |
| AT2G32740 | GT13 | galactosyltransferase 13 |  | 0.07 | 0.01 |
| AT2G33800 |  | Ribosomal protein S5 family protein | STR-THY | 0.24 | 0.02 |
| AT2G34420 | LHCB1.5 | photosystem II light harvesting complex gene B1B2 | THY | 4.19 | 0.31 |
| AT2G34430 | LHCB1.4 | light-harvesting chlorophyll-protein complex II subunit B1 | THY | 36.56 | 2.75 |
| AT2G35170 |  | Histone H3 K4-specific methyltransferase SET7/9 family protein |  | 0.07 | 0.01 |
| AT2G35760 |  | Uncharacterised protein family (UPF0497) |  | 0.34 | 0.03 |
| AT2G37230 |  | Tetratricopeptide repeat (TPR)-like superfamily protein | THY | 0.12 | 0.01 |
| AT2G37620 | ACT1 | actin 1 |  | 0.19 | 0.01 |
| AT2G38040 | CAC3 | acetyl Co-enzyme a carboxylase carboxyltransferase alpha subunit | ENV | 0.04 | 0 |
| AT2G39350 |  | ABC-2 type transporter family protein |  | 0.04 | 0 |
| AT2G39435 |  | Phosphatidylinositol N-acetyglucosaminlytransferase subunit P-related |  | 0.14 | 0.01 |
| AT2G39470 | PNSL1 | PsbP-like protein 2 |  | 1.27 | 0.1 |
| AT2G39730 | RCA | rubisco activase | STR | 0.07 | 0.01 |
| AT2G39795 |  | Mitochondrial glycoprotein family protein |  | 0.13 | 0.01 |
| AT2G42560 |  | late embryogenesis abundant domain-containing protein / LEA domain-containing protein |  | 0.06 | 0 |
| AT2G43030 |  | Ribosomal protein L3 family protein | STR-ENV | 0.13 | 0.01 |
| AT2G43160 |  | ENTH/VHS family protein |  | 0.04 | 0 |
| AT2G43970 |  | RNA-binding protein |  | 0.06 | 0 |
| AT3G01440 | PNSL3 | PsbQ-like 1 | THY | 1.16 | 0.09 |
| AT3G04520 | THA2 | threonine aldolase 2 |  | 0.1 | 0.01 |
| AT3G04910 | WNK1 | with no lysine (K) kinase 1 |  | 0.05 | 0 |
| AT3G07700 |  | Protein kinase superfamily protein |  | 0.05 | 0 |
| AT3G08940 | LHCB4.2 | light harvesting complex photosystem II | THY | 46.62 | 3.5 |
| AT3G11800 |  | unknown protein |  | 0.14 | 0.01 |
| AT3G12345 |  | unknown protein |  | 0.19 | 0.01 |
| AT3G13882 |  | Ribosomal protein L34 |  | 0.17 | 0.01 |
| AT3G14110 | FLU | Tetratricopeptide repeat (TPR)-like superfamily protein | THY | 0.22 | 0.02 |
| AT3G14590 | NTMC2T6.2 | Calcium-dependent lipid-binding (CaLB domain) family protein |  | 0.1 | 0.01 |
| AT3G16000 | MFP1 | MAR binding filament-like protein 1 | THY | 0.33 | 0.02 |
| AT3G16800 |  | Protein phosphatase 2C family protein |  | 0.1 | 0.01 |
| AT3G17570 |  | F-box and associated interaction domains-containing protein |  | 0.08 | 0.01 |
| AT3G18890 |  | NAD(P)-binding Rossmann-fold superfamily protein | THY-ENV | 0.67 | 0.05 |
| AT3G19740 |  | P-loop containing nucleoside triphosphate hydrolases superfamily protein |  | 0.06 | 0 |
| AT3G21430 | ALY3 | DNA binding |  | 0.06 | 0 |
| AT3G22260 |  | Cysteine proteinases superfamily protein |  | 0.14 | 0.01 |
| AT3G22330 | PMH2 | putative mitochondrial RNA helicase 2 |  | 0.12 | 0.01 |
| AT3G24290 | AMT1;5 | ammonium transporter 1;5 |  | 0.07 | 0.01 |
| AT3G25490 |  | Protein kinase family protein |  | 0.08 | 0.01 |
| AT3G25920 | RPL15 | ribosomal protein L15 | STR-ENV | 0.26 | 0.02 |
| AT3G26070 |  | Plastid-lipid associated protein PAP / fibrillin family protein | THY | 0.44 | 0.03 |
| AT3G28370 |  | unknown protein |  | 0.12 | 0.01 |
| AT3G45140 | LOX2 | lipoxygenase 2 | STR | 0.19 | 0.01 |
| AT3G46730 |  | NB-ARC domain-containing disease resistance protein |  | 0.04 | 0 |
| AT3G46780 | PTAC16 | plastid transcriptionally active 16 | THY-ENV | 12.91 | 0.97 |
| AT3G47470 | LHCA4 | light-harvesting chlorophyll-protein complex I subunit A4 | THY | 12.48 | 0.94 |
| AT3G47520 | MDH | malate dehydrogenase | Mix | 0.18 | 0.01 |
| AT3G48870 | ATCLPC | Clp ATPase | ENV-STR | 0.03 | 0 |
| AT3G50820 | PSBO2 | photosystem II subunit O-2 | THY | 2.07 | 0.16 |
| AT3G54890 | LHCA1 | photosystem I light harvesting complex gene 1 | THY | 3.85 | 0.29 |
| AT3G58600 |  | Adaptin ear-binding coat-associated protein 1 NECAP-1 |  | 0.11 | 0.01 |
| AT3G59780 |  | Rhodanese/Cell cycle control phosphatase superfamily protein | THY | 0.18 | 0.01 |
| AT3G61470 | LHCA2 | photosystem I light harvesting complex gene 2 | THY | 1.56 | 0.12 |
| AT3G61480 |  | Quinoprotein amine dehydrogenase, beta chain-like; RIC1-like guanyl-nucleotide exchange factor |  | 0.03 | 0 |
| AT3G61870 |  | unknown protein | THY-ENV | 1.87 | 0.14 |
| AT4G01037 | WTF1 | Ubiquitin carboxyl-terminal hydrolase family protein |  | 0.24 | 0.02 |
| AT4G01050 | TROL | thylakoid rhodanese-like | THY | 4.01 | 0.3 |
| AT4G01150 |  | unknown protein | THY | 0.22 | 0.02 |
| AT4G01690 | PPOX | Flavin containing amine oxidoreductase family | ENV-THY | 0.06 | 0 |
| AT4G02770 | PSAD1 | photosystem I subunit D-1 | THY-ENV | 57.34 | 4.31 |
| AT4G02910 |  | unknown protein |  | 0.27 | 0.02 |
| AT4G03280 | PETC | photosynthetic electron transfer C | THY | 5.98 | 0.45 |
| AT4G04640 | ATPC1 | ATPase, F1 complex, gamma subunit protein | THY-ENV | 35.96 | 2.7 |
| AT4G04910 | NSF | AAA-type ATPase family protein |  | 0.05 | 0 |
| AT4G05180 | PSBQ2 | photosystem II subunit Q-2 | THY | 0.3 | 0.02 |
| AT4G08850 |  | Leucine-rich repeat receptor-like protein kinase family protein |  | 0.03 | 0 |
| AT4G09010 | APX4 | ascorbate peroxidase 4 | THY | 1.6 | 0.12 |
| AT4G09650 | ATPD | ATP synthase delta-subunit gene | THY-ENV | 5.99 | 0.45 |
| AT4G10340 | LHCB5 | light harvesting complex of photosystem II 5 | THY | 47.99 | 3.6 |
| AT4G12800 | PSAL | photosystem I subunit l | THY | 11.93 | 0.9 |
| AT4G13400 |  | 2-oxoglutarate (2OG) and Fe(II)-dependent oxygenase superfamily protein |  | 0.44 | 0.03 |
| AT4G13990 |  | Exostosin family protein |  | 0.06 | 0 |
| AT4G14820 |  | Pentatricopeptide repeat (PPR) superfamily protein |  | 0.04 | 0 |
| AT4G15233 |  | ABC-2 and Plant PDR ABC-type transporter family protein |  | 0.02 | 0 |
| AT4G17250 |  | unknown protein |  | 0.04 | 0 |
| AT4G17600 | LIL3:1 | Chlorophyll A-B binding family protein |  | 0.26 | 0.02 |
| AT4G19010 |  | AMP-dependent synthetase and ligase family protein |  | 0.06 | 0 |
| AT4G20360 | ATRABE1B | RAB GTPase homolog E1B | STR-THY | 0.07 | 0.01 |
| AT4G21280 | PSBQ1 | photosystem II subunit QA | THY | 1.82 | 0.14 |
| AT4G24990 | ATGP4 | Ubiquitin family protein |  | 0.3 | 0.02 |
| AT4G27440 | PORB | protochlorophyllide oxidoreductase B | ENV-THY | 0.99 | 0.07 |
| AT4G28750 | PSAE1 | Photosystem I reaction centre subunit IV / PsaE protein | THY-ENV | 29.09 | 2.18 |
| AT4G29680 |  | Alkaline-phosphatase-like family protein |  | 0.07 | 0.01 |
| AT4G32260 |  | ATPase, F0 complex, subunit B/B', bacterial/chloroplast | THY-ENV | 8.96 | 0.67 |
| AT4G34040 |  | RING/U-box superfamily protein |  | 0.05 | 0 |
| AT4G34240 | ALDH3I | aldehyde dehydrogenase 3I1 | ENV | 0.24 | 0.02 |
| AT4G36390 |  | Methylthiotransferase |  | 0.05 | 0 |
| AT4G38970 | FBA2 | fructose-bisphosphate aldolase 2 | STR | 8.18 | 0.61 |
| AT5G01530 | LHCB4 | light harvesting complex photosystem II | THY | 41.06 | 3.08 |
| AT5G02160 |  | unknown protein |  | 2.03 | 0.15 |
| AT5G04360 | ATLDA | limit dextrinase | STR | 0.09 | 0.01 |
| AT5G05740 | EGY2 | ethylene-dependent gravitropism-deficient and yellow-green-like 2 |  | 0.06 | 0 |
| AT5G10950 |  | Tudor/PWWP/MBT superfamily protein |  | 0.09 | 0.01 |
| AT5G12860 | DiT1 | dicarboxylate transporter 1 | ENV-THY | 0.12 | 0.01 |
| AT5G13120 | PNSL5 | cyclophilin 20-2 | THY | 0.28 | 0.02 |
| AT5G17170 | ENH1 | rubredoxin family protein | THY | 0.78 | 0.06 |
| AT5G17320 | HDG9 | homeodomain GLABROUS 9 |  | 0.05 | 0 |
| AT5G19310 |  | Homeotic gene regulator |  | 0.09 | 0.01 |
| AT5G19940 |  | Plastid-lipid associated protein PAP / fibrillin family protein | ENV-THY | 1.08 | 0.08 |
| AT5G20900 | JAZ12 | jasmonate-zim-domain protein 12 |  | 0.19 | 0.01 |
| AT5G21430 | NDHU | Chaperone DnaJ-domain superfamily protein | ENV-THY | 0.15 | 0.01 |
| AT5G22050 |  | Protein kinase superfamily protein |  | 0.11 | 0.01 |
| AT5G22610 |  | F-box/RNI-like/FBD-like domains-containing protein |  | 0.07 | 0.01 |
| AT5G23060 | CaS | calcium sensing receptor | THY | 4.57 | 0.34 |
| AT5G25070 |  | unknown protein |  | 0.04 | 0 |
| AT5G35170 |  | adenylate kinase family protein | THY | 0.48 | 0.04 |
| AT5G38420 | RBCS-2B | Ribulose bisphosphate carboxylase (small chain) family protein |  | 0.98 | 0.07 |
| AT5G40640 |  | unknown protein |  | 0.06 | 0 |
| AT5G40750 |  | FBD / Leucine Rich Repeat domains containing protein |  | 0.55 | 0.04 |
| AT5G42270 | VAR1 | FtsH extracellular protease family | THY-ENV | 0.69 | 0.05 |
| AT5G42330 |  | unknown protein |  | 0.32 | 0.02 |
| AT5G42650 | AOS | allene oxide synthase | ENV-THY | 1.64 | 0.12 |
| AT5G43750 | PNSB5 | NAD(P)H dehydrogenase 18 |  | 0.64 | 0.05 |
| AT5G44800 | CHR4 | chromatin remodeling 4 |  | 0.01 | 0 |
| AT5G45390 | CLPP4 | CLP protease P4 | STR-ENV | 0.12 | 0.01 |
| AT5G46110 | APE2 | Glucose-6-phosphate/phosphate translocator-related | ENV-THY | 0.8 | 0.06 |
| AT5G47110 | LIL3:2 | Chlorophyll A-B binding family protein |  | 0.13 | 0.01 |
| AT5G48575 |  | Protein of unknown function (DUF1216) |  | 0.35 | 0.03 |
| AT5G49120 |  | Protein of unknown function (DUF581) |  | 0.22 | 0.02 |
| AT5G51570 |  | SPFH/Band 7/PHB domain-containing membrane-associated protein family |  | 0.12 | 0.01 |
| AT5G53800 |  | unknown protein |  | 0.09 | 0.01 |
| AT5G54270 | LHCB3 | light-harvesting chlorophyll B-binding protein 3 | THY | 4.42 | 0.33 |
| AT5G54730 | ATG18F | homolog of yeast autophagy 18 (ATG18) F |  | 0.04 | 0 |
| AT5G56220 |  | P-loop containing nucleoside triphosphate hydrolases superfamily protein |  | 0.03 | 0 |
| AT5G58260 | NDHN | oxidoreductases, acting on NADH or NADPH, quinone or similar compound as acceptor |  | 0.35 | 0.03 |
| AT5G60250 |  | zinc finger (C3HC4-type RING finger) family protein |  | 0.1 | 0.01 |
| AT5G60470 |  | C2H2 and C2HC zinc fingers superfamily protein |  | 0.07 | 0.01 |
| AT5G60520 |  | Late embryogenesis abundant (LEA) protein-related |  | 0.1 | 0.01 |
| AT5G64040 | PSAN | photosystem I reaction center subunit PSI-N, chloroplast, putative / PSI-N, putative (PSAN) | THY | 1.47 | 0.11 |
| AT5G64290 | DIT2.1 | dicarboxylate transport 2.1 | ENV-THY | 0.49 | 0.04 |
| AT5G64910 |  | unknown protein |  | 0.07 | 0.01 |
| AT5G65570 |  | Tetratricopeptide repeat (TPR)-like superfamily protein |  | 0.56 | 0.04 |
| AT5G66190 | FNR1 | ferredoxin-NADP(+)-oxidoreductase 1 | STR-THY | 0.76 | 0.06 |
| AT5G66570 | PSBO1 | PS II oxygen-evolving complex 1 | THY | 3.36 | 0.25 |
| ATCG00020 | PSBA | photosystem II reaction center protein A | THY | 27.53 | 2.07 |
| ATCG00120 | ATPA | ATP synthase subunit alpha | THY-ENV | 26.91 | 2.02 |
| ATCG00130 | ATPF | ATPase, F0 complex, subunit B/B', bacterial/chloroplast | THY | 31.71 | 2.38 |
| ATCG00150 | ATPI | ATPase, F0 complex, subunit A protein | THY | 0.14 | 0.01 |
| ATCG00160 | RPS2 | ribosomal protein S2 | STR | 0.14 | 0.01 |
| ATCG00270 | PSBD | photosystem II reaction center protein D | THY | 17.38 | 1.31 |
| ATCG00280 | PSBC | photosystem II reaction center protein C | THY | 34.45 | 2.59 |
| ATCG00340 | PSAB | Photosystem I, PsaA/PsaB protein | THY-ENV | 7.3 | 0.55 |
| ATCG00350 | PSAA | Photosystem I, PsaA/PsaB protein | THY-ENV | 6.48 | 0.49 |
| ATCG00380 | RPS4 | chloroplast ribosomal protein S4 | STR | 0.16 | 0.01 |
| ATCG00420 | NDHJ | NADH dehydrogenase subunit J |  | 0.21 | 0.02 |
| ATCG00430 | NDHK | photosystem II reaction center protein G |  | 0.83 | 0.06 |
| ATCG00470 | ATPE | ATP synthase epsilon chain | THY-ENV | 10.25 | 0.77 |
| ATCG00480 | ATPB | ATP synthase subunit beta | THY | 154.24 | 11.58 |
| ATCG00490 | RBCL | ribulose-bisphosphate carboxylases | STR-ENV | 7.13 | 0.54 |
| ATCG00500 | ACCD | acetyl-CoA carboxylase carboxyl transferase subunit beta | ENV | 0.07 | 0.01 |
| ATCG00520 | YCF4 | unfolded protein binding |  | 0.72 | 0.05 |
| ATCG00540 | PETA | photosynthetic electron transfer A | THY | 30.08 | 2.26 |
| ATCG00560 | PSBL | photosystem II reaction center protein L | THY | 19.18 | 1.44 |
| ATCG00580 | PSBE | photosystem II reaction center protein E | THY | 12.51 | 0.94 |
| ATCG00650 | RPS18 | ribosomal protein S18 | ENV-STR | 0.33 | 0.02 |
| ATCG00660 | RPL20 | ribosomal protein L20 | ENV-STR | 0.28 | 0.02 |
| ATCG00680 | PSBB | photosystem II reaction center protein B | THY | 41.02 | 3.08 |
| ATCG00710 | PSBH | photosystem II reaction center protein H | THY | 14.7 | 1.1 |
| ATCG00720 | PETB | photosynthetic electron transfer B | THY | 5.91 | 0.44 |
| ATCG00730 | PETD | photosynthetic electron transfer D | THY | 2.43 | 0.18 |
| ATCG00750 | RPS11 | ribosomal protein S11 | STR | 1.51 | 0.11 |
| ATCG00770 | RPS8 | ribosomal protein S8 | STR-ENV | 1.06 | 0.08 |
| ATCG00800 | RPS3 | structural constituent of ribosome | STR-ENV | 1.22 | 0.09 |
| ATCG00830 | RPL2.1 | ribosomal protein L2 | STR-ENV | 0.13 | 0.01 |
| ATCG01010 | NDHF | NADH-Ubiquinone oxidoreductase (complex I), chain 5 protein |  | 0.04 | 0 |
| ATCG01060 | PSAC | iron-sulfur cluster binding;electron carriers;4 iron, 4 sulfur cluster binding | THY | 140.16 | 10.53 |
| ATCG01070 | NDHE | NADH-ubiquinone/plastoquinone oxidoreductase chain 4L | THY | 3.15 | 0.24 |
| ATCG01090 | NDHI | NADPH dehydrogenases |  | 1.55 | 0.12 |
| ATCG01110 | NDHH | NAD(P)H dehydrogenase subunit H | STR-THY | 0.17 | 0.01 |

Localization of each protein was followed the data in the AT_CHLORO database (Ferro et al. 2010). The emPAI value of each protein showed a sum of emPAI values in all the gel slices from the BN-gel. The PC (mol%) is the protein content in molar percentages, which was calculated according to the calculation formula described by Ishihama et al. 2005.
