# Supplementary material for: Protein co-migration database (PCoM -DB) for Arabidopsis thylakoids and Synechocystis cells
Source: Springerplus. 2013 Apr 8;2:148. doi: 10.1186/2193-1801-2-148 (PMC3647082; doi:10.1186/2193-1801-2-148)
Supplement: Supplementary file 3 — Additional file 3: Figure S1: Protein migration profiles of the remaining subunits of the PSII, PSI, and ATP synthase proteins. The emPAI-based protein migration profiles of the remaining PSII core subunits (PsbE, PsbH, and PsbL) (A), the remaining PSI proteins (PsaE, PsaF, PsaG,PsaH2, PsaK, PsaL, and PsaN) (B), and the remaining ATP synthase subunits (AtpE, AtpF, AtpI, and AT4G32260) (C). (PDF 452 KB) [file 40064_2013_228_MOESM3_ESM.pdf]

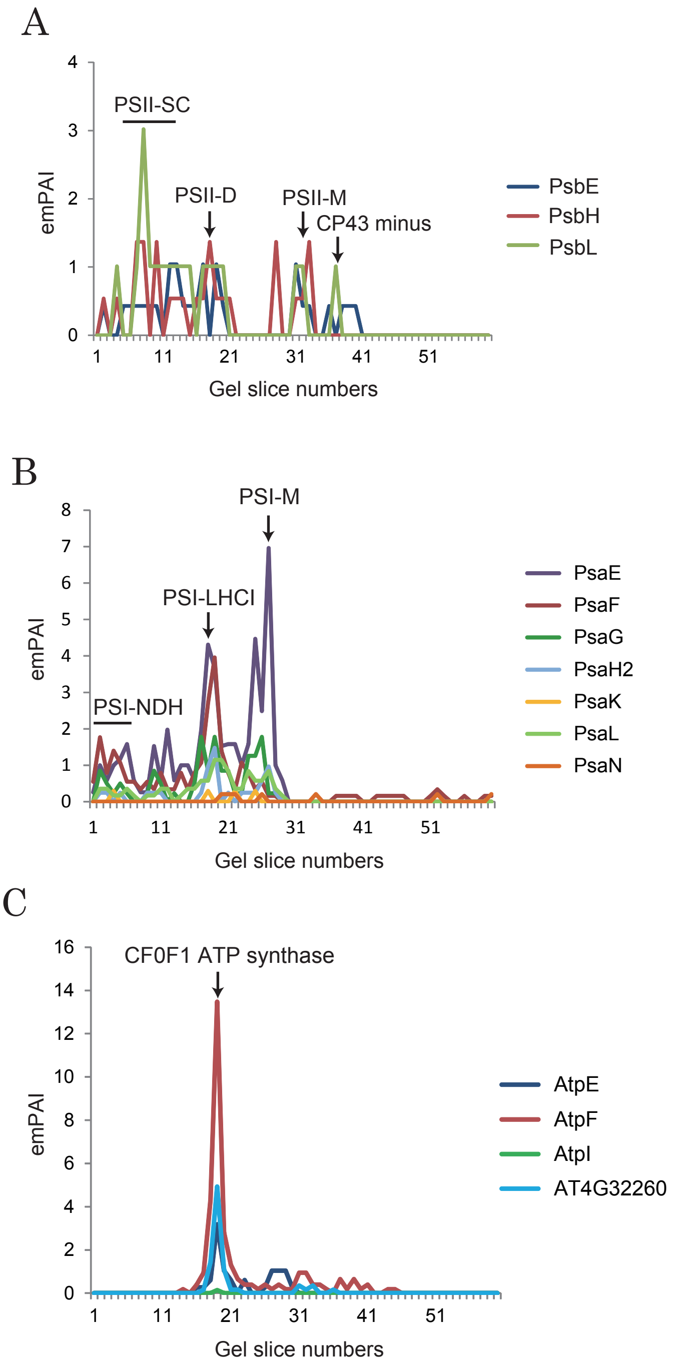

Additional file 3 . Protein migration profiles of the remaining subunits of the PSII, PSI, and ATP synthase proteins. The emPAI-based protein migration profiles of the remaining PSII core subunits (PsbE, PsbH, and PsbL) (A), the remaining PSI proteins (PsaE, PsaF, PsaG, PsaH2, PsaK, PsaL, and PsaN) (B), and the remaining ATP synthase subunits (AtpE, AtpF, AtpI, and AT4G32260) (C).
