# Supplementary material for: Protein co-migration database (PCoM -DB) for Arabidopsis thylakoids and Synechocystis cells
Source: Springerplus. 2013 Apr 8;2:148. doi: 10.1186/2193-1801-2-148 (PMC3647082; doi:10.1186/2193-1801-2-148)
Supplement: Supplementary file 4 — Additional file 4: Figure S2: Protein migration profiles of the remaining subunits of the NDH proteins. The emPAI-based protein migration profiles of the six NDH proteins (NdhE, NdhF, NdhL, NdhN, NdhU, and PnsB1) (A) and the remaining NDH proteins (PnsB2, PnsB5, PnsL5, PnsL1, PnsL2, and PnsL3) (B). (PDF 364 KB) [file 40064_2013_228_MOESM4_ESM.pdf]

A

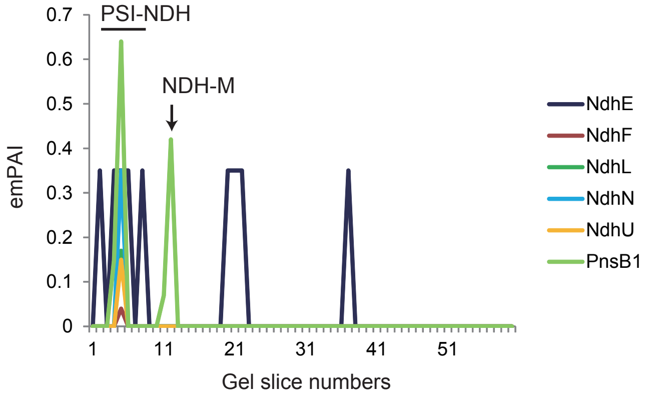

B

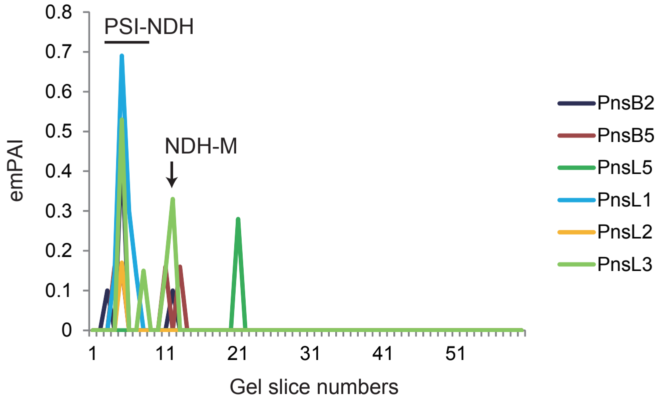

Additional file 4 . Protein migration profiles of the remaining subunits of the NDH proteins. The emPAI-based protein migration profiles of the six NDH proteins (NdhE, NdhF, NdhL, NdhN, NdhU, and PnsB1) (A) and the remaining NDH proteins (PnsB2, PnsB5, PnsL5, PnsL1, PnsL2, and PnsL3) (B).
