# Supplementary material for: Protein co-migration database (PCoM -DB) for Arabidopsis thylakoids and Synechocystis cells
Source: Springerplus. 2013 Apr 8;2:148. doi: 10.1186/2193-1801-2-148 (PMC3647082; doi:10.1186/2193-1801-2-148)
Supplement: Supplementary file 5 — Additional file 5: Table S3: Identified proteins from Synechocystis cells. (DOC 1 MB) [file 40064_2013_228_MOESM5_ESM.doc]

**Additional File 5: Table S3.** Identified proteins from *Synechocystis* cells

| Locus | Symbols | Description | emPAI |
| --- | --- | --- | --- |
| sll0002 |  | penicillin-binding protein | 0.12 |
| sll0005 |  | hypothetical protein | 0.05 |
| sll0006 |  | putative aminotransferase | 0.39 |
| sll0017 | hemL | glutamate-1-semialdehyde aminomutase | 2.77 |
| sll0018 | fbaA | fructose-bisphosphate aldolase, class II | 46.29 |
| sll0019 |  | 1-deoxy-d-xylulose 5-phosphate reductoisomerase | 1.22 |
| sll0020 |  | ATP-dependent Clp protease ATPase subunit | 13.36 |
| sll0023 |  | hypothetical protein | 0.32 |
| sll0027 | ndhD4 | NADH dehydrogenase subunit 4 (involved in constitutive, low affinity CO2 uptake) | 0.21 |
| sll0033 | crtH | carotene isomerase | 0.54 |
| sll0036 |  | hypothetical protein | 0.16 |
| sll0038 | pixG | positive phototaxis protein, two-component response regulator PatA subfamily | 0.85 |
| sll0041 | pixJ1 | phytochrome-like photoreceptor protein for positive phototaxis; homologous to methyl-accepting chemotaxis protein | 7.69 |
| sll0042 | pixJ2 | methyl-accepting chemotaxis protein for positive phototaxis | 1.62 |
| sll0043 | pixL | positive phototaxis protein, homologous to chemotaxis protein CheA, two-component hybrid histidine kinase | 0.37 |
| sll0048 |  | unknown protein | 1.06 |
| sll0051 |  | hypothetical protein | 9.67 |
| sll0053 | accC | biotin carboxylase | 0.4 |
| sll0057 | grpE | heat shock protein GrpE | 4.7 |
| sll0058 | dnaK1 | DnaK protein 1, heat shock protein 70, molecular chaperone | 1.16 |
| sll0064 |  | periplasmic protein, putative polar amino acid transport system substrate-binding protein | 0.8 |
| sll0065 | ilvN | acetolactate synthase small subunit | 2.18 |
| sll0067 |  | glutathione S-transferase | 0.19 |
| sll0068 |  | unknown protein | 0.03 |
| sll0069 |  | hypothetical protein | 2.99 |
| sll0071 |  | hypothetical protein | 0.1 |
| sll0078 | thrS | threonyl-tRNA synthetase | 0.33 |
| sll0080 | argC | N-acetyl-gamma-glutamyl-phosphate reductase | 2.83 |
| sll0086 |  | putative arsenical pump-driving ATPase | 1.41 |
| sll0095 |  | hypothetical protein | 0.06 |
| sll0096 |  | hypothetical protein | 0.28 |
| sll0098 |  | hypothetical protein | 0.19 |
| sll0102 |  | hypothetical protein | 5.01 |
| sll0103 |  | hypothetical protein | 2.1 |
| sll0135 |  | putative 5'-methylthioadenosine phosphorylase | 1.13 |
| sll0136 |  | aminopeptidase P | 0.34 |
| sll0141 |  | hypothetical protein | 0.07 |
| sll0144 | pyrH | uridine monophosphate kinase | 3.13 |
| sll0145 | frr | ribosome releasing factor | 2.21 |
| sll0146 | natC | Integral membrane protein of the ABC-type, Nat permease for neutral amino acids | 0.19 |
| sll0147 |  | hypothetical protein | 0.05 |
| sll0148 |  | hypothetical protein | 0.25 |
| sll0149 |  | hypothetical protein | 0.06 |
| sll0158 | glgB | 1,4-alpha-glucan branching enzyme | 2.97 |
| sll0162 |  | hypothetical protein | 0.17 |
| sll0163 |  | WD-repeat protein | 0.74 |
| sll0166 | hemD | a fusion protein between uroporphyrinogen-III C-methyltransferase (CobA/CorA) and uroporphyrinogen-III synthase (HemD) | 0.12 |
| sll0170 | dnaK2 | DnaK protein 2, heat shock protein 70, molecular chaperone | 14.02 |
| sll0171 |  | probable aminomethyltransferase | 0.49 |
| sll0173 | vgb | virginiamycin B hydrolase, periplasmic protein | 2.4 |
| sll0178 |  | hypothetical protein | 0.02 |
| sll0179 | gltX | glutamyl-tRNA synthetase | 2.33 |
| sll0180 |  | hypothetical protein | 15.54 |
| sll0185 |  | hypothetical protein | 3.64 |
| sll0195 |  | probable ATP-dependent protease | 0.15 |
| sll0199 | petE | plastocyanin | 22.46 |
| sll0204 |  | glucose inhibited division protein | 0.07 |
| sll0207 | rfbA | glucose-1-phosphate thymidylyltransferase | 0.18 |
| sll0209 |  | hypothetical protein | 0.42 |
| sll0210 |  | bacitracin resistance protein | 0.11 |
| sll0217 |  | flavoprotein | 5.57 |
| sll0218 |  | hypothetical protein | 1.43 |
| sll0219 |  | flavoprotein | 10.86 |
| sll0220 | glmS | L-glutamine:D-fructose-6-P amidotransferase | 0.74 |
| sll0221 |  | bacterioferritin comigratory protein | 1.18 |
| sll0223 | ndhB | NADH dehydrogenase subunit 2 | 2.18 |
| sll0224 |  | amino-acid ABC transporter binding protein | 0.38 |
| sll0226 | ycf4 | photosystem I assembly related protein | 4.11 |
| sll0227 | ppiB | peptidyl-prolyl cis-trans isomerase B, periplasmic protein | 3.67 |
| sll0228 | speB1 | arginase | 0.24 |
| sll0230 |  | hypothetical protein | 19.01 |
| sll0236 |  | unknown protein | 2.71 |
| sll0241 |  | unknown protein | 0.1 |
| sll0243 |  | unknown protein | 0.24 |
| sll0245 |  | probable GTP binding protein | 2.02 |
| sll0247 | isiA | iron-stress chlorophyll-binding protein, homologous to psbC (CP43) | 13.51 |
| sll0248 | isiB | flavodoxin | 0 |
| sll0250 |  | pantothenate metabolism flavoprotein | 0.92 |
| sll0254 |  | probable phytoene dehydrogenase Rieske iron-sulfur component | 0.05 |
| sll0258 | psbV | cytochrome c550 | 15.99 |
| sll0267 |  | unknown protein | 0.04 |
| sll0271 |  | N utilization substance protein B homolog | 0.71 |
| sll0272 |  | hypothetical protein | 9.65 |
| sll0274 |  | hypothetical protein | 0.36 |
| sll0283 |  | hypothetical protein | 2.49 |
| sll0289 | minD | septum site-determining protein MinD | 3.35 |
| sll0290 | ppk | polyphosphate kinase | 0.17 |
| sll0294 |  | hypothetical protein | 0.08 |
| sll0300 | ribC | riboflavin synthase alpha chain | 0.33 |
| sll0301 |  | hypothetical protein | 0.9 |
| sll0309 |  | unknown protein | 0.14 |
| sll0312 |  | probable oligopeptides ABC transporter permease protein | 0.1 |
| sll0314 |  | periplasmic protein, function unknown | 1.24 |
| sll0318 |  | hypothetical protein | 0.16 |
| sll0320 |  | probable ribonuclease D | 9.3 |
| sll0321 |  | unknown protein | 0.07 |
| sll0325 |  | hypothetical protein | 0.21 |
| sll0329 |  | 6-phosphogluconate dehydrogenase | 19.26 |
| sll0330 |  | sepiapterine reductase | 0.13 |
| sll0337 | phoR | phosphate sensor, two-component sensor histidine kinase | 0.08 |
| sll0359 |  | hypothetical protein | 21.55 |
| sll0362 | alaS | alanyl-tRNA synthetase | 0.6 |
| sll0368 |  | uracil phosphoribosyltransferase | 0.19 |
| sll0370 | pyrA | carbamoyl-phosphate synthase, pyrimidine-specific, large chain | 2.08 |
| sll0372 |  | hypothetical protein | 0.17 |
| sll0373 | proA | gamma-glutamyl phosphate reductase | 0.67 |
| sll0374 | urtE | urea transport system ATP-binding protein | 0.42 |
| sll0379 |  | acyl-[acyl-carrier-protein]--UDP-N-acetylglucosamine o-acyltransferase | 0.36 |
| sll0395 |  | phosphoglycerate mutase | 3.56 |
| sll0401 |  | citrate synthase | 8.21 |
| sll0402 | aspC | aspartate aminotransferase | 1.32 |
| sll0404 | glcD | glycolate oxidase subunit GlcD | 0.21 |
| sll0406 |  | unknown protein | 0.21 |
| sll0408 |  | peptidyl-prolyl cis-trans isomerase | 3.27 |
| sll0410 |  | hypothetical protein | 0.2 |
| sll0412 |  | hypothetical protein | 0.16 |
| sll0413 |  | hypothetical protein | 0.48 |
| sll0414 |  | hypothetical protein | 0.72 |
| sll0415 |  | ATP-binding protein of ABC transporter | 0.27 |
| sll0416 | groEL-2 | 60 kDa chaperonin 2, GroEL2, molecular chaperone | 79.73 |
| sll0418 |  | 2-methyl-6-phytylbenzoquinone methyltransferase | 0.11 |
| sll0420 | ureB | urease beta subunit | 0.35 |
| sll0421 | purB | adenylosuccinate lyase | 7.35 |
| sll0422 |  | asparaginase | 0.89 |
| sll0427 | psbO | photosystem II manganese-stabilizing polypeptide | 11.48 |
| sll0430 | htpG | HtpG, heat shock protein 90, molecular chaperone | 0.21 |
| sll0443 |  | unknown protein | 0.37 |
| sll0445 |  | unknown protein | 0.07 |
| sll0446 |  | unknown protein | 0.76 |
| sll0450 | norB | cytochrome b subunit of nitric oxide reductase | 0.14 |
| sll0451 |  | hypothetical protein | 0.22 |
| sll0454 | pheS | phenylalanyl-tRNA synthetase alpha chain | 3.34 |
| sll0455 | thrA | homoserine dehydrogenase | 0.35 |
| sll0456 |  | hypothetical protein | 0.1 |
| sll0461 | proA | gamma-glutamyl phosphate reductase | 4.73 |
| sll0467 |  | S-adenosylmethionine:tRNA ribosyltransferase-isomerase | 0.09 |
| sll0469 |  | ribose-phosphate pyrophosphokinase | 4.32 |
| sll0470 |  | hypothetical protein | 0.58 |
| sll0474 | hik28 | two-component hybrid sensor and regulator | 0.04 |
| sll0477 |  | putative biopolymer transport ExbB-like protein | 0.13 |
| sll0480 |  | probable aminotransferase | 10.32 |
| sll0487 |  | hypothetical protein | 0.33 |
| sll0489 |  | ATP-binding protein of ABC transporter | 0.2 |
| sll0493 |  | hypothetical protein | 1.04 |
| sll0495 | asnS | asparaginyl-tRNA synthetase | 1.13 |
| sll0497 |  | hypothetical protein | 0.24 |
| sll0502 | argS | arginyl-tRNA-synthetase | 0.36 |
| sll0504 | lysA | diaminopimelate decarboxylase | 0.52 |
| sll0505 |  | hypothetical protein | 0.22 |
| sll0507 |  | probable cation transporter | 0.37 |
| sll0518 |  | unknown protein | 1.32 |
| sll0519 | ndhA | NADH dehydrogenase subunit 1 | 7.1 |
| sll0520 | ndhI | NADH dehydrogenase subunit NdhI | 249.05 |
| sll0521 | ndhG | NADH dehydrogenase subunit 6 | 12.13 |
| sll0522 | ndhE | NADH dehydrogenase subunit 4L | 7.8 |
| sll0524 |  | hypothetical protein | 0.25 |
| sll0529 |  | hypothetical protein | 4 |
| sll0533 |  | trigger factor | 13.92 |
| sll0534 | clpP2 | ATP-dependent Clp protease proteolytic subunit 2 | 1.24 |
| sll0542 |  | acetyl-coenzyme A synthetase | 1.28 |
| sll0550 |  | flavoprotein | 11.51 |
| sll0553 |  | hypothetical protein | 0.85 |
| sll0554 | ftrC | ferredoxin-thioredoxin reductase, catalytic chain | 3.14 |
| sll0558 | ycf53 | hypothetical protein YCF53 | 0.14 |
| sll0567 | fur | ferric uptake regulation protein | 1.04 |
| sll0569 | recA | RecA gene product | 3.94 |
| sll0572 |  | hypothetical protein | 0.07 |
| sll0574 |  | probable permease protein of lipopolysaccharide ABC transporter | 0.24 |
| sll0575 |  | probable lipopolysaccharide ABC transporter ATP binding subunit | 0.13 |
| sll0576 |  | putative sugar-nucleotide epimerase/dehydratease | 8.13 |
| sll0577 |  | hypothetical protein | 0.21 |
| sll0578 | purK | phosphoribosylaminoimidazole carboxylase ATPase subunit | 0.09 |
| sll0585 |  | hypothetical protein | 0.4 |
| sll0586 |  | hypothetical protein | 0.08 |
| sll0587 |  | pyruvate kinase | 0.14 |
| sll0588 |  | unknown protein | 0.69 |
| sll0593 |  | glucokinase | 0.52 |
| sll0596 |  | hypothetical protein | 1.56 |
| sll0601 |  | nitrilase homolog | 0.54 |
| sll0602 |  | hypothetical protein | 0.95 |
| sll0606 |  | hypothetical protein | 0.07 |
| sll0616 | secA | preprotein translocase SecA subunit | 7.55 |
| sll0617 | vipp1 | plasma membrane protein essential for thylakoid formation | 7.76 |
| sll0625 |  | unknown protein | 1.31 |
| sll0626 |  | putative neutral invertase | 0.07 |
| sll0629 | psaK2 | alternative photosystem I reaction center subunit X | 9.86 |
| sll0631 | nadB | L-aspartate oxidase | 0.31 |
| sll0634 | btpA | photosystem I biogenesis protein BtpA | 1.28 |
| sll0635 | thiE | probable thiamine-phosphate pyrophosphorylase | 0.1 |
| sll0638 |  | periplasmic protein, function unknown | 0.07 |
| sll0639 |  | hypothetical protein | 0.32 |
| sll0641 |  | unknown protein | 0.06 |
| sll0644 |  | probable esterase | 0.2 |
| sll0657 |  | phospho-N-acetylmuramoyl-pentapeptide-transferase | 0.09 |
| sll0661 | ycf35 | hypothetical protein YCF35 | 1.88 |
| sll0672 | pacL | cation-transporting p-type ATPase PacL | 0.99 |
| sll0679 |  | periplasmic phosphate-binding protein of ABC transporter | 0.2 |
| sll0680 |  | phosphate-binding periplasmic protein precursor (PBP) | 1.05 |
| sll0685 |  | hypothetical protein | 0.22 |
| sll0688 |  | unknown protein | 0.13 |
| sll0689 | nhaS3 | Na+/H+ antiporter | 1.6 |
| sll0708 |  | dimethyladenosine transferase | 0.12 |
| sll0712 | cysM | cysteine synthase | 1.56 |
| sll0726 |  | phosphoglucomutase | 2.93 |
| sll0735 |  | hypothetical protein | 6.14 |
| sll0736 |  | hypothetical protein | 0.08 |
| sll0741 |  | pyruvate flavodoxin oxidoreductase | 1.12 |
| sll0744 |  | hypothetical protein | 1.06 |
| sll0749 |  | hypothetical protein | 0.18 |
| sll0752 |  | hypothetical protein | 1 |
| sll0753 | folD | FolD bifunctional protein | 1.11 |
| sll0754 |  | ribosome binding factor A | 0.26 |
| sll0755 | tpx | thioredoxin peroxidase | 0.87 |
| sll0756 |  | unknown protein | 0.54 |
| sll0757 | purF | amidophosphoribosyltransferase | 0.63 |
| sll0759 |  | ABC transporter ATP-binding protein | 0.3 |
| sll0764 | urtD | urea transport system ATP-binding protein | 0.09 |
| sll0767 | rpl20 | 50S ribosomal protein L20 | 70.14 |
| sll0771 | glcP | glucose transport protein | 0.24 |
| sll0776 | spkD | serine/threonine kinase | 0.07 |
| sll0781 |  | hypothetical protein | 3.97 |
| sll0783 |  | unknown protein | 0.22 |
| sll0784 | merR | nitrilase | 0.1 |
| sll0787 |  | hypothetical protein | 0.11 |
| sll0788 |  | hypothetical protein | 1.06 |
| sll0807 | rpe | pentose-5-phosphate-3-epimerase | 8.15 |
| sll0813 | ctaC | cytochrome c oxidase subunit II | 0.88 |
| sll0815 |  | unknown protein | 3.39 |
| sll0816 |  | probable oxidoreductase | 0.3 |
| sll0819 | psaF | photosystem I reaction center subunit III precursor (PSI-F), plastocyanin (cyt c553) docking protein | 1553.6 |
| sll0822 |  | hypothetical protein | 2.48 |
| sll0827 |  | hypothetical protein | 0.12 |
| sll0828 |  | putative amidase | 0.57 |
| sll0830 | fus | elongation factor EF-G | 1.71 |
| sll0833 |  | probable oligopeptides ABC transporter permease protein | 0.19 |
| sll0837 |  | periplasmic protein, function unknown | 1.56 |
| sll0838 | pyrF | orotidine 5' monophosphate decarboxylase | 0.15 |
| sll0848 | dnaA | chromosomal replication initiator protein DnaA | 0.07 |
| sll0849 | psbD | photosystem II reaction center D2 protein | 21.41 |
| sll0851 | psbC | photosystem II CP43 protein | 33.24 |
| sll0853 |  | hypothetical protein | 2.24 |
| sll0854 |  | hypothetical protein | 0.62 |
| sll0860 |  | hypothetical protein | 0.44 |
| sll0861 |  | hypothetical protein | 0.12 |
| sll0871 |  | hypothetical protein | 1.44 |
| sll0873 |  | carboxynorspermidine decarboxylase | 0.09 |
| sll0877 |  | hypothetical protein | 0.07 |
| sll0887 |  | putative modulator of DNA gyrase; PmbA homolog | 1.29 |
| sll0891 |  | malate dehydrogenase | 0.55 |
| sll0895 |  | CysQ protein homolog | 0.56 |
| sll0897 | dnaJ | DnaJ protein, heat shock protein 40, molecular chaperone | 0.09 |
| sll0899 |  | UDP-N-acetylglucosamine pyrophosphorylase | 1.68 |
| sll0900 | hisG | ATP phosphoribosyltransferase | 0.32 |
| sll0901 | purE | phosphoribosylaminoimidazole carboxylase | 0.97 |
| sll0902 | argF | ornithine carbamoyltransferase | 2.48 |
| sll0905 |  | hypothetical protein | 0.18 |
| sll0909 |  | unknown protein | 0.14 |
| sll0910 |  | unknown protein | 0.09 |
| sll0912 |  | ABC transporter ATP binding protein | 0.05 |
| sll0914 |  | unknown protein | 0.09 |
| sll0915 |  | periplasmic protease | 0.2 |
| sll0920 | ppc | phosphoenolpyruvate carboxylase | 1.21 |
| sll0921 |  | two-component response regulator NarL subfamily | 0.32 |
| sll0923 |  | unknown protein | 1.13 |
| sll0924 |  | hypothetical protein | 0.18 |
| sll0927 |  | S-adenosylmethionine synthetase | 2.4 |
| sll0928 | apcD | allophycocyanin-B | 175.43 |
| sll0931 |  | hypothetical protein | 0.23 |
| sll0932 |  | hypothetical protein | 0.1 |
| sll0934 | ccmA | carboxysome formation protein CcmA | 2.98 |
| sll0936 |  | putative oxidoreductase | 0.28 |
| sll0938 |  | aspartate transaminase | 0.09 |
| sll0945 | glgA | glycogen synthase | 1.49 |
| sll0947 | lrtA | light repressed protein A homolog | 25.54 |
| sll0982 |  | unknown protein | 2.97 |
| sll0985 |  | unknown protein | 0.05 |
| sll0990 |  | glutathione-dependent formaldehyde dehydrogenase | 0.47 |
| sll0992 |  | putative esterase | 0.28 |
| sll0993 |  | potassium channel | 0.09 |
| sll0995 |  | hypothetical protein | 0.45 |
| sll0997 |  | hypothetical protein | 0.42 |
| sll0998 | ycf30 | LysR family transcriptional regulator | 6.08 |
| sll1004 |  | hypothetical protein | 0.62 |
| sll1018 | pyrC | dihydroorotase | 0.69 |
| sll1019 |  | hydroxyacylglutathione hydrolase | 0.39 |
| sll1020 |  | probable glycosyltransferase | 0.77 |
| sll1021 |  | hypothetical protein | 10.4 |
| sll1023 |  | succinyl-CoA synthetase beta chain | 0.16 |
| sll1027 | gltD | NADH-dependent glutamate synthase small subunit | 3.97 |
| sll1028 | ccmK2 | carbon dioxide concentrating mechanism protein CcmK | 158.16 |
| sll1029 | ccmK1 | carbon dioxide concentrating mechanism protein CcmK | 235.41 |
| sll1030 | ccmL | carbon dioxide concentrating mechanism protein CcmL, putative carboxysome assembly protein | 0.36 |
| sll1031 | ccmM | carbon dioxide concentrating mechanism protein CcmM, putative carboxysome structural protein | 1.54 |
| sll1033 |  | probable protein phosphatase | 0.56 |
| sll1035 |  | uracil phosphoribosyltransferase | 2.17 |
| sll1043 |  | polyribonucleotide nucleotidyltransferase | 31.67 |
| sll1051 | cpcF | phycocyanin alpha-subunit phycocyanobilin lyase | 0.16 |
| sll1053 |  | hypothetical protein | 0.7 |
| sll1054 |  | hypothetical protein | 0.4 |
| sll1056 | purL | phosphoribosylformyl glycinamidine synthetase II | 0.75 |
| sll1058 | dapB | dihydrodipicolinate reductase | 4.42 |
| sll1059 |  | adenylate kinase | 1.83 |
| sll1060 |  | hypothetical protein | 0.15 |
| sll1064 |  | hypothetical protein | 0.18 |
| sll1069 |  | 3-oxoacyl-[acyl-carrier-protein] synthase II | 1.25 |
| sll1070 |  | transketolase | 12.35 |
| sll1074 | leuS | leucyl-tRNA synthetase | 1.41 |
| sll1076 | pacL | cation-transporting ATPase PacL | 0.06 |
| sll1077 | speB2 | agmatinase | 0.28 |
| sll1089 |  | periplasmic protein, function unknown | 1.64 |
| sll1091 | chlP | geranylgeranyl hydrogenase | 1.16 |
| sll1096 | rps12 | 30S ribosomal protein S12 | 15.32 |
| sll1097 | rps7 | 30S ribosomal protein S7 | 181.07 |
| sll1098 | fus | elongation factor EF-G | 2.56 |
| sll1099 | tufA | elongation factor Tu | 575.54 |
| sll1101 | rps10 | 30S ribosomal protein S10 | 29.84 |
| sll1106 |  | hypothetical protein | 28.97 |
| sll1108 |  | stationary-phase survival protein SurE homolog | 0.12 |
| sll1109 |  | hypothetical protein | 0.93 |
| sll1110 | prfA | peptide chain release factor 1 | 0.6 |
| sll1118 |  | hypothetical protein | 8.3 |
| sll1121 |  | hypothetical protein | 0.12 |
| sll1124 | plpA | two-component sensor histidine kinase, phytochrome-like protein | 0.02 |
| sll1127 | menB | 1,4-dihydroxy-2-naphthoate synthase | 1.75 |
| sll1130 |  | unknown protein | 230.4 |
| sll1135 |  | unknown protein | 0.1 |
| sll1138 |  | hypothetical protein | 0.14 |
| sll1143 | pcrA | ATP-dependent helicase PcrA | 0.04 |
| sll1151 |  | unknown protein | 0.06 |
| sll1154 |  | putative antibiotic efflux protein | 0.08 |
| sll1158 |  | hypothetical protein | 0.27 |
| sll1162 |  | hypothetical protein | 0.14 |
| sll1165 |  | DNA mismatch repair protein | 0.04 |
| sll1166 |  | hypothetical protein | 0.09 |
| sll1170 |  | unknown protein | 0.06 |
| sll1172 | thrC | threonine synthase | 0.09 |
| sll1174 |  | unknown protein | 0.12 |
| sll1180 |  | toxin secretion ABC transporter ATP-binding protein | 0.06 |
| sll1181 |  | similar to hemolysin secretion protein | 0.06 |
| sll1184 | ho1 | heme oxygenase | 0.44 |
| sll1185 | hemF | coproporphyrinogen III oxidase, aerobic (oxygen-dependent) | 1.6 |
| sll1188 |  | hypothetical protein | 2.05 |
| sll1194 | psbU | photosystem II 12 kDa extrinsic protein | 8.77 |
| sll1196 |  | phosphofructokinase | 0.2 |
| sll1198 |  | tRNA (guanine-N1)-methyltransferase | 0.15 |
| sll1201 |  | hypothetical protein | 0.27 |
| sll1212 |  | GDP-mannose 4,6-dehydratase | 7.95 |
| sll1213 |  | GDP-fucose synthetase | 6.03 |
| sll1214 | ycf59 | hypothetical protein YCF59 | 0.27 |
| sll1217 |  | unknown protein | 0.3 |
| sll1218 | ycf39 | hypothetical protein YCF39 | 2.74 |
| sll1220 | hoxE | putative diaphorase subunit of the bidirectional hydrogenase | 0.2 |
| sll1221 | hoxF | diaphorase subunit of the bidirectional hydrogenase | 0.37 |
| sll1223 | hoxU | diaphorase subunit of the bidirectional hydrogenase | 0.48 |
| sll1226 | hoxH | hydrogenase subunit of the bidirectional hydrogenase | 0.54 |
| sll1228 | hik4 | two-component hybrid sensor and regulator | 0.16 |
| sll1233 |  | hypothetical protein | 0.22 |
| sll1234 |  | adenosylhomocysteinase | 19.51 |
| sll1236 |  | unknown protein | 0.26 |
| sll1239 |  | unknown protein | 0.36 |
| sll1242 |  | hypothetical protein | 0.37 |
| sll1244 | rpl9 | 50S ribosomal protein L9 | 35.14 |
| sll1247 |  | hypothetical protein | 0.07 |
| sll1249 | panC | pantothenate synthetase/cytidylate kinase | 0.07 |
| sll1252 |  | hypothetical protein | 0.41 |
| sll1253 |  | similar to polyA polymerase | 0.06 |
| sll1254 |  | hypothetical protein | 0.1 |
| sll1258 |  | dCTP deaminase | 0.36 |
| sll1260 | rps2 | 30S ribosomal protein S2 | 46.46 |
| sll1261 | tsf | elongation factor TS | 13.43 |
| sll1262 |  | hypothetical protein | 88.97 |
| sll1268 |  | unknown protein | 0.07 |
| sll1270 | bgtB | periplasmic substrate-binding and integral membrane protein of the ABC-type Bgt permease for basic amino acids and glutamine BgtB | 1.41 |
| sll1272 |  | unknown protein | 1.45 |
| sll1275 |  | pyruvate kinase 2 | 5.46 |
| sll1276 |  | ATP-binding protein of ABC transporter | 0.12 |
| sll1282 | ribH | riboflavin synthase beta subunit | 5.92 |
| sll1284 |  | esterase | 0.17 |
| sll1285 |  | hypothetical protein | 0.44 |
| sll1290 |  | probable ribonuclease II | 0.05 |
| sll1291 |  | two-component response regulator PatA subfamily | 0.41 |
| sll1292 |  | two-component response regulator CheY subfamily | 2.27 |
| sll1294 |  | methyl-accepting chemotaxis protein | 3.91 |
| sll1296 | hik39 | two-component hybrid sensor and regulator | 0.08 |
| sll1298 |  | putative carboxymethylenebutenolidase | 1.76 |
| sll1299 |  | acetate kinase | 0.17 |
| sll1304 |  | unknown protein | 0.46 |
| sll1305 |  | probable hydrolase | 1.24 |
| sll1306 |  | periplasmic protein, function unknown | 13.26 |
| sll1307 |  | periplasmic protein, function unknown | 7.15 |
| sll1308 |  | probable oxidoreductase | 0.77 |
| sll1314 |  | putative C4-dicarboxylase binding protein, periplasmic protein | 2.04 |
| sll1315 |  | unknown protein | 0.44 |
| sll1316 | petC1 | cytochrome b6-f complex iron-sulfur subunit (Rieske iron sulfur protein) | 4.43 |
| sll1317 | petA | apocytochrome f, component of cytochrome b6/f complex | 15.23 |
| sll1318 |  | hypothetical protein | 0.3 |
| sll1322 | atpI | ATP synthase A chain of CF(0) | 1.91 |
| sll1323 | atpG | ATP synthase subunit b' of CF(0) | 14.74 |
| sll1324 | atpF | ATP synthase B chain (subunit I) of CF(0) | 118.91 |
| sll1325 | atpD | ATP synthase delta chain of CF(1) | 37.43 |
| sll1326 | atpA | ATP synthase alpha chain | 87.44 |
| sll1327 | atpC | ATP synthase gamma chain | 19.5 |
| sll1334 |  | two-component sensor histidine kinase | 3.1 |
| sll1336 |  | hypothetical protein | 5.74 |
| sll1341 |  | bacterioferritin | 225.04 |
| sll1342 | gap2 | NAD(P)-dependent glyceraldehyde-3-phosphate dehydrogenase | 35.78 |
| sll1343 |  | aminopeptidase | 5.85 |
| sll1348 |  | hypothetical protein | 0.11 |
| sll1349 |  | phosphoglycolate phosphatase | 0.15 |
| sll1350 |  | hypothetical protein | 0.08 |
| sll1356 |  | glycogen phosphorylase | 3.99 |
| sll1358 |  | putative oxalate decarboxylase, periplasmic protein | 2.32 |
| sll1362 | ileS | isoleucyl-tRNA synthetase | 0.54 |
| sll1363 | ilvC | ketol-acid reductoisomerase | 109.31 |
| sll1365 |  | unknown protein | 0.05 |
| sll1370 | rfbM | mannose-1-phosphate guanylyltransferase | 0.18 |
| sll1380 |  | periplasmic protein, function unknown | 2.04 |
| sll1381 |  | hypothetical protein | 0.25 |
| sll1384 |  | similar to DnaJ protein | 0.22 |
| sll1388 |  | hypothetical protein | 1.2 |
| sll1390 |  | hypothetical protein | 5.49 |
| sll1393 | glgA | glycogen (starch) synthase | 1.26 |
| sll1395 | rfbD | dTDP-6-deoxy-L-mannose-dehydrogenase | 0.12 |
| sll1397 |  | putative transposase [ISY100a: 52234 - 53180] | 0.11 |
| sll1398 | psb28, psbW, psb13, ycf79 | photosystem II reaction center 13 kDa protein | 1.92 |
| sll1404 |  | biopolymer transport ExbB protein homolog | 0.17 |
| sll1411 |  | hypothetical protein | 0.23 |
| sll1414 |  | hypothetical protein | 7.06 |
| sll1415 |  | hypothetical protein | 0.75 |
| sll1418 | psbP2 | photosystem II oxygen-evolving complex 23K protein PsbP homolog | 2.58 |
| sll1423 | ntcA | global nitrogen regulator | 2.73 |
| sll1425 | proS | proline-tRNA ligase | 3.57 |
| sll1426 |  | unknown protein | 0.46 |
| sll1430 |  | adenine phosphoribosyltransferase | 0.93 |
| sll1433 |  | hypothetical protein | 2.69 |
| sll1435 |  | glutamyl-tRNA(Gln) amidotransferase subunit B | 3.57 |
| sll1440 | pdxH | pyridoxamine 5'-phosphate oxidase | 0.6 |
| sll1443 | pyrG | CTP synthetase | 5.21 |
| sll1444 |  | 3-isopropylmalate dehydratase small subunit | 0.8 |
| sll1446 |  | hypothetical protein | 0.11 |
| sll1450 | nrtA | nitrate/nitrite transport system substrate-binding protein | 10.77 |
| sll1452 | nrtC | nitrate/nitrite transport system ATP-binding protein | 0.1 |
| sll1455 |  | hypothetical protein | 0.22 |
| sll1456 |  | unknown protein | 3.48 |
| sll1457 |  | probable glycosyltransferase | 0.59 |
| sll1459 |  | stationary-phase survival protein SurE homolog | 0.15 |
| sll1463 | ftsH | cell division protein FtsH | 7.87 |
| sll1464 |  | hypothetical protein | 0.14 |
| sll1466 |  | probable glycosyltransferase | 0.16 |
| sll1470 | leuC | 3-isopropylmalate dehydratase large subunit | 0.64 |
| sll1471 | cpcG2 | phycobilisome rod-core linker polypeptide | 46.87 |
| sll1473 |  | a part of phytochrome-like sensor histidine kinase gene (disrupted by insertion of IS) | 0.07 |
| sll1479 |  | 6-phosphogluconolactonase | 3.45 |
| sll1481 |  | ABC-transporter membrane fusion protein | 0.24 |
| sll1484 | ndbC | type 2 NADH dehydrogenase | 1.8 |
| sll1489 | cpmA | circadian phase modifier CpmA homolog | 0.4 |
| sll1491 |  | periplasmic WD-repeat protein | 0.54 |
| sll1496 |  | mannose-1-phosphate guanyltransferase | 0.04 |
| sll1498 |  | carbamoyl-phosphate synthase small chain | 0.29 |
| sll1499 | glsF | ferredoxin-dependent glutamate synthase | 2.13 |
| sll1502 | gltB | NADH-dependent glutamate synthase large subunit | 5.89 |
| sll1509 | ycf20 | hypothetical protein YCF20 | 0.31 |
| sll1513 | ccsA | c-type cytochrome synthesis protein | 0.1 |
| sll1514 | hspA | 16.6 kDa small heat shock protein, molecular chaperone | 0.23 |
| sll1520 | recN | DNA repair protein RecN | 0.12 |
| sll1521 |  | flavoprotein | 2.1 |
| sll1525 | prk | phosphoribulokinase | 41.23 |
| sll1526 |  | hypothetical protein | 0.69 |
| sll1527 |  | unknown protein | 0.06 |
| sll1528 |  | unknown protein | 0.29 |
| sll1530 |  | unknown protein | 0.54 |
| sll1533 | pilT2 | twitching mobility protein | 1.08 |
| sll1534 |  | probable glycosyltransferase | 0.09 |
| sll1535 |  | putative sugar transferase | 0.14 |
| sll1536 | moeB | molybdopterin biosynthesis MoeB protein | 19.1 |
| sll1537 |  | similar to mutator MutT protein | 2.49 |
| sll1538 |  | similar to beta-hexosaminidase a precursor | 0.06 |
| sll1541 |  | hypothetical protein | 0.07 |
| sll1545 |  | glutathione S-transferase | 1.88 |
| sll1549 |  | salt-enhanced periplasmic protein | 0.14 |
| sll1550 |  | probable porin; major outer membrane protein | 0.12 |
| sll1553 | pheT | phenylalanyl-tRNA synthetase | 5.86 |
| sll1557 |  | succinyl-CoA synthetase alpha chain | 0.12 |
| sll1558 |  | mannose-1-phosphate guanyltransferase | 0.36 |
| sll1559 |  | soluble hydrogenase 42 kD subunit | 18.63 |
| sll1561 | putA | proline oxidase | 3.39 |
| sll1562 |  | unknown protein | 0.22 |
| sll1563 |  | unknown protein | 0.13 |
| sll1566 | ggpS | glucosylglycerolphosphate synthase | 1.05 |
| sll1568 |  | fibrillin | 0.36 |
| sll1577 | cpcB | phycocyanin beta subunit | 3134 |
| sll1578 | cpcA | phycocyanin alpha subunit | 3728.7 |
| sll1579 | cpcC2 | phycobilisome rod linker polypeptide | 263.11 |
| sll1580 | cpcC1 | phycobilisome rod linker polypeptide | 284.44 |
| sll1581 |  | hypothetical protein | 0.21 |
| sll1583 |  | unknown protein | 15.38 |
| sll1594 | ndhR | ndhF3 operon transcriptional regulator, LysR family protein | 9.5 |
| sll1595 | kaiC2 | circadian clock protein KaiC homolog | 0.12 |
| sll1608 |  | hypothetical protein | 0.12 |
| sll1612 | folC | folylpolyglutamate synthase | 0.08 |
| sll1614 | pma1 | cation-transporting P-type ATPase | 0.04 |
| sll1618 |  | hypothetical protein | 1.11 |
| sll1620 |  | hypothetical protein | 0.22 |
| sll1621 |  | AhpC/TSA family protein | 111.59 |
| sll1624 |  | two-component response regulator | 0.1 |
| sll1625 |  | succinate dehydrogenase iron- sulphur protein subunit | 0.51 |
| sll1626 |  | LexA repressor | 53.62 |
| sll1633 | ftsZ | cell division protein FtsZ | 6.78 |
| sll1635 |  | Thy1 protein homolog | 0.32 |
| sll1636 |  | ferripyochelin binding protein | 0.2 |
| sll1638 |  | hypothetical protein | 20.78 |
| sll1639 | ureD | urease accessory protein D | 0.14 |
| sll1640 |  | hypothetical protein | 0.44 |
| sll1641 |  | glutamate decarboxylase | 8.08 |
| sll1654 |  | hypothetical protein | 2.88 |
| sll1655 |  | similar to biotin [acetyl-CoA-carboxylase] ligase | 0.13 |
| sll1656 |  | hypothetical protein | 1.77 |
| sll1660 |  | hypothetical protein | 0.68 |
| sll1662 |  | probable prephenate dehydratase | 0.12 |
| sll1663 |  | phycocyanin alpha phycocyanobilin lyase related protein | 3.04 |
| sll1665 |  | unknown protein | 4.13 |
| sll1667 |  | periplasmic protein, similar to mitochondrial outer membrane 72K protein | 0.13 |
| sll1672 | hik12 | two-component hybrid sensor and regulator | 0.04 |
| sll1675 |  | hypothetical protein | 0.24 |
| sll1676 |  | 4-alpha-glucanotransferase | 0.21 |
| sll1677 |  | similar to spore maturation protein B | 0.34 |
| sll1679 | hhoA | periplasmic protease HhoA | 0.28 |
| sll1680 |  | hypothetical protein | 0.19 |
| sll1682 |  | alanine dehydrogenase | 5.07 |
| sll1683 |  | lysine decarboxylase | 0.07 |
| sll1687 | hik17 | unknown protein | 0.35 |
| sll1688 | thrC | threonine synthase | 0.24 |
| sll1693 |  | hypothetical protein | 2.32 |
| sll1694 | pilA1 | pilin polypeptide PilA1 | 44.47 |
| sll1696 |  | hypothetical protein | 0.42 |
| sll1697 |  | hypothetical protein | 0.4 |
| sll1699 |  | oligopeptide-binding protein of oligopeptide ABC transporter | 0.66 |
| sll1702 | ycf51 | hypothetical protein YCF51 | 0.2 |
| sll1703 |  | protease IV | 0.24 |
| sll1709 |  | 3-ketoacyl-acyl carrier protein reductase | 0.47 |
| sll1712 |  | DNA binding protein HU | 143.85 |
| sll1721 |  | pyruvate dehydrogenase E1 component, beta subunit | 6.85 |
| sll1732 | ndhF3 | NADH dehydrogenase subunit 5 (involved in low CO2-inducible, high affinity CO2 uptake) | 0.24 |
| sll1733 | ndhD3 | NADH dehydrogenase subunit 4 (involved in low CO2-inducible, high affinity CO2 uptake) | 1.62 |
| sll1734 | cupA | protein involved in low CO2-inducible, high affinity CO2 uptake | 24.76 |
| sll1735 |  | hypothetical protein | 13.24 |
| sll1740 | rpl19 | 50S ribosomal protein L19 | 18.85 |
| sll1742 | nusG | transcription antitermination protein NusG | 0.16 |
| sll1743 | rpl11 | 50S ribosomal protein L11 | 16.09 |
| sll1744 | rpl1 | 50S ribosomal protein L1 | 36.34 |
| sll1745 | rpl10 | 50S ribosomal protein L10 | 10.61 |
| sll1746 | rpl12 | 50S ribosomal protein L12 | 36.78 |
| sll1747 | aroC | chorismate synthase | 3.83 |
| sll1750 | ureC | urease alpha subunit | 0.44 |
| sll1757 |  | hypothetical protein | 6.64 |
| sll1760 | thrB | homoserine kinase | 0.12 |
| sll1762 |  | periplasmic protein, putative polar amino acid transport system substrate-binding protein | 0.69 |
| sll1767 | rps6 | 30S ribosomal protein S6 | 11.87 |
| sll1768 |  | probable oligopeptides ABC transporter permease protein | 0.1 |
| sll1771 | pphA | protein serin-threonin phosphatase | 1.44 |
| sll1775 |  | hypothetical protein | 0.3 |
| sll1776 |  | deoxyribose-phosphate aldolase | 0.5 |
| sll1783 |  | hypothetical protein | 1.37 |
| sll1784 |  | periplasmic protein, function unknown | 0.4 |
| sll1785 |  | periplasmic protein, function unknown | 2.17 |
| sll1786 | tatD | putative deoxyribonuclease, tatD homolog | 0.4 |
| sll1787 | rpoB | RNA polymerase beta subunit | 25.8 |
| sll1789 | rpoC2 | RNA polymerase beta prime subunit | 29.55 |
| sll1799 | rpl3 | 50S ribosomal protein L3 | 44.39 |
| sll1800 | rpl4 | 50S ribosomal protein L4 | 25.55 |
| sll1801 | rpl23 | 50S ribosomal protein L23 | 33.61 |
| sll1802 | rpl2 | 50S ribosomal protein L2 | 66.24 |
| sll1803 | rpl22 | 50S ribosomal protein L22 | 29.75 |
| sll1804 | rps3 | 30S ribosomal protein S3 | 51.95 |
| sll1805 | rpl16 | 50S ribosomal protein L16 | 26.99 |
| sll1806 | rpl14 | 50S ribosomal protein L14 | 34.79 |
| sll1807 | rpl24 | 50S ribosomal protein L24 | 52.89 |
| sll1808 | rpl5 | 50S ribosomal protein L5 | 37.73 |
| sll1809 | rps8 | 30S ribosomal protein S8 | 121.33 |
| sll1810 | rpl6 | 50S ribosomal protein L6 | 17.51 |
| sll1811 | rpl18 | 50S ribosomal protein L18 | 40.18 |
| sll1812 | rps5 | 30S ribosomal protein S5 | 341.62 |
| sll1813 | rpl15 | 50S ribosomal protein L15 | 42.2 |
| sll1814 | secY | preprotein translocase SecY subunit | 0.16 |
| sll1815 | adk | adenylate kinase | 18.31 |
| sll1816 | rps13 | 30S ribosomal protein S13 | 38.13 |
| sll1817 | rps11 | 30S ribosomal protein S11 | 69.74 |
| sll1818 | rpoA | RNA polymerase alpha subunit | 64.87 |
| sll1819 | rpl17 | 50S ribosomal protein L17 | 54.42 |
| sll1821 | rpl13 | 50S ribosomal protein L13 | 24.26 |
| sll1822 | rps9 | 30S ribosomal protein S9 | 22.85 |
| sll1823 | purA | adenylosuccinate synthetase | 1.45 |
| sll1824 | rpl25 | 50S ribosomal protein L25 | 3.2 |
| sll1825 |  | hypothetical protein | 3.68 |
| sll1830 |  | unknown protein | 3.25 |
| sll1831 |  | glycolate oxidase subunit, (Fe-S)protein | 0.15 |
| sll1833 |  | penicillin-binding protein | 0.06 |
| sll1834 |  | hypothetical protein | 0.25 |
| sll1835 |  | periplasmic protein, function unknown | 2.61 |
| sll1837 |  | periplasmic protein, function unknown | 0.81 |
| sll1841 |  | pyruvate dehydrogenase dihydrolipoamide acetyltransferase component (E2) | 26.96 |
| sll1852 |  | nucleoside diphosphate kinase | 24.46 |
| sll1871 | hik6 | two-component system sensory histidine kinase | 0.15 |
| sll1873 |  | unknown protein | 14.99 |
| sll1879 | ycf55 | two-component response regulator | 0.12 |
| sll1882 |  | unknown protein | 0.24 |
| sll1883 | argJ | arginine biosynthesis bifunctional protein ArgJ | 2.26 |
| sll1888 | hik5 | two-component sensor histidine kinase | 0.08 |
| sll1891 |  | unknown protein | 0.42 |
| sll1892 |  | unknown protein | 0.15 |
| sll1893 | hisF | cyclase | 0.42 |
| sll1894 | ribA | riboflavin biosynthesis protein RibA | 0.06 |
| sll1895 |  | hypothetical protein | 0.2 |
| sll1897 |  | hypothetical protein | 1.5 |
| sll1899 | ctaB | cytochrome c oxidase folding protein | 0.33 |
| sll1908 | serA | D-3-phosphoglycerate dehydrogenase | 17.77 |
| sll1910 | zam | protein conferring resistance to acetazolamide Zam | 0.12 |
| sll1913 |  | hypothetical protein | 0.18 |
| sll1927 |  | ABC transporter ATP-binding protein | 0.2 |
| sll1929 | comE | competence protein ComE | 0.05 |
| sll1931 | glyA | serine hydroxymethyltransferase | 1.43 |
| sll1932 | dnaK | DnaK protein | 0.66 |
| sll1937 |  | ferric uptake regulation protein | 0.25 |
| sll1939 |  | unknown protein | 0.16 |
| sll1941 | gyrA | DNA gyrase A subunit | 0.04 |
| sll1945 |  | 1-deoxyxylulose-5-phosphate synthase | 1.55 |
| sll1956 |  | hypothetical protein | 0.17 |
| sll1958 | hisC | histidinol phosphate aminotransferase | 0.55 |
| sll1960 |  | hypothetical protein | 0.75 |
| sll1961 |  | hypothetical protein | 0.18 |
| sll1967 |  | probable RNA methyltransferase | 0.07 |
| sll1979 |  | hypothetical protein | 4.14 |
| sll1981 | ilvB | acetolactate synthase | 0.46 |
| sll1987 | katG | catalase peroxidase | 0.95 |
| sll1994 | hemB | porphobilinogen synthase (5-aminolevulinate dehydratase) | 19.48 |
| sll2001 |  | leucine aminopeptidase | 8.34 |
| sll2002 |  | hypothetical protein | 0.66 |
| sll2005 | gyrB | DNA gyrase B subunit [Contains: Ssp gyrB intein] | 0.54 |
| sll2009 |  | processing protease | 0.08 |
| sll2010 | murD | UDP-N-acetylmuramoylalanine--D-glutamate ligase | 0.16 |
| sll2014 |  | sugar fermentation stimulation protein | 0.28 |
| sll2015 |  | hypothetical protein | 0.16 |
| sll5034 |  | hypothetical protein | 0.35 |
| sll5044 |  | unknown protein | 0.16 |
| sll5052 |  | similar to exopolysaccharide export protein | 0.08 |
| sll5059 |  | two-component response regulator | 0.53 |
| sll5066 |  | probable plasmid partitioning protein, ParA family | 0.85 |
| sll5067 |  | hypothetical protein | 0.19 |
| sll5075 |  | hypothetical protein | 1.05 |
| sll5080 |  | non-heme chloroperoxidase | 1.03 |
| sll5123 |  | SOS mutagenesis and repair, UmuD protein homolog | 0.24 |
| sll5128 |  | unknown protein | 0.4 |
| sll6060 |  | probable acetyltransferase | 0.2 |
| sll7030 |  | hypothetical protein | 0.2 |
| sll7043 |  | unknown protein | 0.36 |
| sll7044 |  | plasmid partition protein ParA homolog | 0.18 |
| sll7065 |  | unknown protein | 0.07 |
| sll7075 |  | unknown protein | 0.09 |
| sll8019 |  | hypothetical protein | 0.06 |
| slr0001 |  | hypothetical protein | 2.23 |
| slr0006 |  | unknown protein | 31.18 |
| slr0007 |  | probable sugar-phosphate nucleotidyltransferase | 0.28 |
| slr0009 | rbcL | ribulose bisphosphate carboxylase large subunit | 1240.8 |
| slr0012 | rbcS | ribulose bisphosphate carboxylase small subunit | 96.57 |
| slr0013 |  | hypothetical protein | 15.06 |
| slr0014 |  | Mg2+ transport ATPase | 0.15 |
| slr0015 |  | lipid A disaccharide synthase | 0.81 |
| slr0017 | murA | UDP-N-acetylglucosamine 1-carboxyvinyltransferase | 0.08 |
| slr0018 |  | fumarase | 0.38 |
| slr0020 | recG | DNA recombinase | 0.04 |
| slr0021 |  | protease | 2.38 |
| slr0031 |  | hypothetical protein | 1.48 |
| slr0032 |  | probable branched-chain amino acid aminotransferase | 9.04 |
| slr0033 |  | glutamyl-tRNA(Gln) amidotransferase subunit C | 0.34 |
| slr0038 |  | hypothetical protein | 5.57 |
| slr0039 |  | hypothetical protein | 1.26 |
| slr0040 | cmpA | bicarbonate transport system substrate-binding protein | 110.04 |
| slr0041 | cmpB | bicarbonate transport system permease protein | 2.27 |
| slr0042 |  | probable porin; major outer membrane protein | 0.3 |
| slr0043 | cmpC | bicarbonate transport system ATP-binding protein | 3.4 |
| slr0044 | cmpD | bicarbonate transport system ATP-binding protein | 2.19 |
| slr0049 |  | hypothetical protein | 2.13 |
| slr0050 | ycf56 | hypothetical protein YCF56 | 0.1 |
| slr0051 | ecaB | periplasmic beta-type carbonic anhydrase | 1.14 |
| slr0053 |  | hypothetical protein | 0.57 |
| slr0063 | pilB1 | pilus biogenesis protein homologous to general secretion pathway protein E | 0.1 |
| slr0065 |  | hypothetical protein | 0.16 |
| slr0066 | ribD | riboflavin biosynthesis protein RibD | 0.18 |
| slr0067 |  | MRP protein homolog | 2.1 |
| slr0070 | fmt | methionyl-tRNA formyltransferase | 0.58 |
| slr0072 | gidB | glucose inhibited division protein B | 0.41 |
| slr0073 | hik36 | two-component sensor histidine kinase | 0.95 |
| slr0074 | ycf24 | ABC transporter subunit | 0.14 |
| slr0075 | ycf16 | ABC transporter ATP-binding protein | 1 |
| slr0079 |  | probable general secretion pathway protein E | 0.74 |
| slr0080 | rnhA | ribonuclease H | 0.21 |
| slr0084 | hisH | amidotransferase HisH | 0.17 |
| slr0086 |  | similar to DnaK protein | 0.42 |
| slr0088 | crtO | beta-carotene ketolase | 0.5 |
| slr0089 |  | gamma-tocopherol methyltransferase | 0.3 |
| slr0106 |  | unknown protein | 0.45 |
| slr0108 |  | unknown protein | 0.08 |
| slr0110 |  | hypothetical protein | 0.51 |
| slr0115 | rpaA | response regulator for energy transfer from phycobilisomes to photosystems | 2.44 |
| slr0116 | pcyA | phycocyanobilin:ferredoxin oxidoreductase | 1.32 |
| slr0118 | thiC | thiamine biosynthesis protein ThiC | 0.97 |
| slr0143 | hat | WD-repeat protein, Hat protein, involved in the control of a high-affinity transport system for inorganic carbon | 0.03 |
| slr0147 |  | hypothetical protein | 0.24 |
| slr0148 |  | hypothetical protein | 2.64 |
| slr0149 |  | hypothetical protein | 0.42 |
| slr0151 |  | unknown protein | 2.01 |
| slr0156 | clpB2 | ClpB protein | 0.45 |
| slr0161 | pilT1 | twitching motility protein PilT | 3 |
| slr0162 | pilC | a part of pilC, pilin biogenesis protein, required for twitching motility | 0.84 |
| slr0163 | pilC' | a part of pilC, pilin biogenesis protein, required for twitching motility | 0.12 |
| slr0164 | clpP4 | ATP-dependent Clp protease proteolytic subunit | 9.09 |
| slr0165 | clpP3 | ATP-dependent Clp protease proteolytic subunit | 17.72 |
| slr0169 |  | hypothetical protein | 0.87 |
| slr0171 | ycf37 | photosystem I assembly related protein Ycf37 | 0.57 |
| slr0172 |  | hypothetical protein | 3.95 |
| slr0185 |  | orotate phosphoribosyltransferase | 0.18 |
| slr0186 | leuA | 2-isopropylmalate synthase | 1.05 |
| slr0191 |  | amidase enhancer, periplasmic protein | 0.25 |
| slr0193 | rbp3 | RNA-binding protein | 5.3 |
| slr0194 | rpiA | ribose 5-phosphate isomerase | 0.54 |
| slr0201 |  | heterodisulfide reductase subunit B | 0.44 |
| slr0209 |  | unknown protein | 0.49 |
| slr0212 | metH | 5-methyltetrahydrofolate--homocysteine methyltransferase | 0.72 |
| slr0213 | guaA | GMP synthetase | 5.16 |
| slr0214 |  | cytosine-specific methyltransferase(5'-CGATCG-3') | 0.16 |
| slr0220 | glyS | glycyl-tRNA synthetase beta chain | 2.22 |
| slr0226 |  | unknown protein | 0.07 |
| slr0228 | ftsH | cell division protein FtsH | 4.48 |
| slr0229 |  | 3-hydroxyisobutyrate dehydrogenase | 0.27 |
| slr0237 |  | glycogen operon protein GlgX homolog | 0.22 |
| slr0242 |  | bacterioferritin comigratory protein homolog | 0.22 |
| slr0244 |  | hypothetical protein | 10.92 |
| slr0251 | ycf85 | ATP-binding protein of ABC transporter | 0.57 |
| slr0252 |  | probable precorrin-6x reductase | 0.39 |
| slr0260 | cobO | cob(I)alamin adenosyltransferase | 0.54 |
| slr0261 | ndhH | NADH dehydrogenase subunit 7 | 138.98 |
| slr0280 |  | hypothetical protein | 0.12 |
| slr0286 |  | protein involved in functional assembly of photosystem II | 0.07 |
| slr0287 |  | hypothetical protein | 0.66 |
| slr0288 | glnN | glutamate--ammonia ligase | 0.2 |
| slr0293 |  | glycine dehydrogenase | 0.63 |
| slr0298 |  | FraH protein homolog | 0.17 |
| slr0301 |  | phosphoenolpyruvate synthase | 1.11 |
| slr0302 |  | unknown protein | 0.04 |
| slr0315 |  | probable oxidoreductase | 1.37 |
| slr0317 |  | hypothetical protein | 0.12 |
| slr0320 |  | hypothetical protein | 0.06 |
| slr0322 | hik43 | two-component hybrid sensor and regulator | 0.86 |
| slr0323 |  | putative alpha-mannosidase | 0.03 |
| slr0328 |  | low molecular weight phosphotyrosine protein phosphatase | 0.44 |
| slr0331 | ndhD1 | NADH dehydrogenase subunit 4 (involved in photosystem-1 cyclic electron flow) | 0.38 |
| slr0335 | apcE | phycobilisome core-membrane linker polypeptide | 282.58 |
| slr0338 |  | probable oxidoreductase | 0.09 |
| slr0341 |  | unknown protein | 0.11 |
| slr0342 | petB | cytochrome b6 | 9.08 |
| slr0343 | petD | cytochrome b6-f complex subunit 4 | 8.05 |
| slr0344 |  | probable glycosyltransferase | 0.07 |
| slr0348 |  | hypothetical protein | 0.48 |
| slr0353 |  | unknown protein | 1.46 |
| slr0355 |  | hypothetical protein | 0.35 |
| slr0357 | hisS | histidyl-tRNA synthetase | 0.08 |
| slr0359 |  | hypothetical protein | 0.03 |
| slr0361 |  | probable ribosomal large subunit pseudouridine synthase B | 0.13 |
| slr0369 |  | RND multidrug efflux transporter | 2.65 |
| slr0370 |  | succinate-semialdehyde dehydrogenase (NADP+) | 1.82 |
| slr0374 |  | hypothetical protein | 2.96 |
| slr0376 |  | hypothetical protein | 5.02 |
| slr0380 |  | hypothetical protein | 0.3 |
| slr0387 | nifS | cysteine desulfurase NifS | 0.19 |
| slr0388 |  | hypothetical protein | 0.15 |
| slr0394 | pgk | phosphoglycerate kinase | 17.53 |
| slr0397 |  | hypothetical protein | 0.18 |
| slr0398 |  | unknown protein | 0.42 |
| slr0399 | ycf39 | chaperon-like protein for quinone binding in photosystem II | 2.21 |
| slr0400 |  | hypothetical protein | 0.11 |
| slr0401 |  | periplasmic polyamine-binding protein of ABC transporter | 0.09 |
| slr0402 |  | hypothetical protein | 0.18 |
| slr0404 |  | hypothetical protein | 2.61 |
| slr0406 | pyrC | dihydroorotase | 0.2 |
| slr0417 | gyrA | DNA gyrase subunit A | 3.62 |
| slr0426 | folE | GTP cyclohydrolase I | 8.37 |
| slr0427 |  | putative competence-damage protein | 0.17 |
| slr0431 |  | hypothetical protein | 0.58 |
| slr0434 | efp | elongation factor P | 5.1 |
| slr0435 | accB | biotin carboxyl carrier protein of acetyl-CoA carboxylase | 0.24 |
| slr0438 |  | hypothetical protein | 0.7 |
| slr0440 |  | hypothetical protein | 0.12 |
| slr0442 |  | unknown protein | 0.65 |
| slr0444 | aroA | 3-phosphoshikimate 1-carboxyvinyltransferase | 0.42 |
| slr0447 | urtA | periplasmic protein, ABC-type urea transport system substrate-binding protein | 24.53 |
| slr0452 | ilvD | dihydroxyacid dehydratase | 4.95 |
| slr0453 |  | hypothetical protein | 3.08 |
| slr0454 |  | RND multidrug efflux transporter | 0.04 |
| slr0455 |  | hypothetical protein | 1.19 |
| slr0458 |  | unknown protein | 0.16 |
| slr0467 | natA | conserved component of ABC transporter for natural amino acids | 0.97 |
| slr0468 |  | unknown protein | 0.07 |
| slr0469 | rps4 | 30S ribosomal protein S4 | 95.11 |
| slr0473 | cph1 | cyanobacterial phytochrome 1, two-component sensor histidine kinase | 0.17 |
| slr0476 |  | unknown protein | 14.35 |
| slr0477 | purN | phosphoribosylglycinamide formyltransferase | 0.16 |
| slr0480 | ycf46 | hypothetical protein YCF46 | 0.42 |
| slr0482 |  | unknown protein | 0.13 |
| slr0483 |  | hypothetical protein | 42.97 |
| slr0484 | hik26 | two-component sensor histidine kinase | 0.05 |
| slr0488 |  | virulence factor MviN homolog. | 0.07 |
| slr0498 |  | unknown protein | 0.28 |
| slr0500 | hisB | imidazoleglycerol-phosphate dehydratase | 2.07 |
| slr0502 | cobW | cobalamin synthesis protein cobW homolog | 0.1 |
| slr0503 | ycf66 | hypothetical protein YCF66 | 1.13 |
| slr0506 | por | light-dependent NADPH-protochlorophyllide oxidoreductase | 5.67 |
| slr0510 |  | hypothetical protein | 0.12 |
| slr0513 |  | iron transport system substrate-binding protein, periplasmic protein | 20.47 |
| slr0520 | purL | phosphoribosyl formylglycinamidine synthase | 0.45 |
| slr0521 |  | unknown protein | 0.1 |
| slr0525 | chlM | Mg-protoporphyrin IX methyl transferase | 0.15 |
| slr0526 | panB | 3-methyl-2-oxobutanoate hydroxymethyltransferase | 2.66 |
| slr0527 |  | transcription regulator ExsB homolog | 0.13 |
| slr0528 | murE | UDP-N-acetylmuramoylalanyl-D-glutamate--2, 6-diaminopimelate ligase | 0.46 |
| slr0534 |  | probable transglycosylase | 0.04 |
| slr0535 |  | protease | 0.06 |
| slr0536 | hemE | uroporphyrinogen decarboxylase | 2.53 |
| slr0537 |  | putative sugar kinase | 0.33 |
| slr0542 | clpP | ATP-dependent protease ClpP | 3.66 |
| slr0543 | trpB | tryptophan synthase beta subunit | 2.02 |
| slr0545 |  | hypothetical protein | 0.11 |
| slr0549 | asd | aspartate beta-semialdehyde dehydrogenese | 1.62 |
| slr0550 | dapA | dihydrodipicolinate synthase | 0.99 |
| slr0552 |  | hypothetical protein | 7.66 |
| slr0557 | valS | valyl-tRNA synthetase | 0.58 |
| slr0559 | natB | periplasmic binding protein of ABC transporter for natural amino acids | 3.41 |
| slr0575 |  | hypothetical protein | 1.17 |
| slr0583 |  | similar to GDP-fucose synthetase | 0.33 |
| slr0585 | argG | argininosuccinate synthetase | 14.44 |
| slr0586 |  | hypothetical protein | 0.13 |
| slr0596 |  | hypothetical protein | 0.42 |
| slr0597 | purH | phosphoribosyl aminoimidazole carboxy formyl formyltransferase/inosinemonophosphate cyclohydrolase (PUR-H(J)) | 3.04 |
| slr0599 |  | serine/threonine kinase | 0.13 |
| slr0600 |  | NADP-thioredoxin reductase | 0.1 |
| slr0603 | dnaE | DNA polymerase III alpha subunit [Contains: Ssp dnaE intein] | 0.04 |
| slr0605 |  | hypothetical protein | 1.13 |
| slr0606 |  | hypothetical protein | 1.31 |
| slr0607 |  | hypothetical protein | 1.61 |
| slr0609 |  | hypothetical protein | 0.09 |
| slr0612 |  | probable pseudouridine synthase | 0.18 |
| slr0613 |  | hypothetical protein | 0.47 |
| slr0619 |  | hypothetical protein | 0.55 |
| slr0623 | trxA | thioredoxin | 20.74 |
| slr0624 |  | UDP-N-acetylglucosamine 2-epimerase | 0.82 |
| slr0628 | rps14 | 30S ribosomal protein S14 | 22.28 |
| slr0633 | thiG | thiamine biosynthesis protein ThiG | 0.32 |
| slr0635 |  | hypothetical protein | 0.94 |
| slr0637 |  | hypothetical protein | 4.35 |
| slr0638 | glyQ | glycyl-tRNA synthetase alpha chain | 0.59 |
| slr0645 |  | hypothetical protein | 4.77 |
| slr0646 |  | probable D-alanyl-D-alanine carboxypeptidase | 0.3 |
| slr0649 | metS | methionyl-tRNA synthetase | 0.51 |
| slr0650 |  | hypothetical protein | 7.9 |
| slr0651 |  | hypothetical protein | 0.56 |
| slr0652 | hisA | phosphorybosilformimino-5-amino- phosphorybosil-4-imidazolecarboxamideisomerase | 1.26 |
| slr0653 | sigA | principal RNA polymerase sigma factor SigA | 1.04 |
| slr0654 |  | unknown protein | 0.28 |
| slr0657 |  | aspartate kinase | 0.78 |
| slr0659 |  | oligopeptidase A | 1.57 |
| slr0661 | proC | pyrroline-5-carboxylate reductase | 2.07 |
| slr0662 |  | arginine decarboxylase | 0.05 |
| slr0665 |  | aconitate hydratase | 1.89 |
| slr0666 |  | unknown protein | 1.13 |
| slr0670 |  | hypothetical protein | 7.43 |
| slr0676 | cysC | adenylylsulfate kinase | 4.33 |
| slr0677 |  | biopolymer transport ExbB like protein | 1.82 |
| slr0678 |  | biopolymer transport ExbD like protein | 2.09 |
| slr0680 |  | hypothetical protein | 0.5 |
| slr0688 |  | hypothetical protein | 0.1 |
| slr0689 |  | hypothetical protein | 0.67 |
| slr0692 | ycf45 | hypothetical protein YCF45 | 0.29 |
| slr0695 |  | hypothetical protein | 0.64 |
| slr0702 |  | unknown protein | 0.08 |
| slr0707 | polA | DNA polymerase I | 0.03 |
| slr0708 |  | periplasmic protein, function unknown | 4.17 |
| slr0709 |  | hypothetical protein | 1.28 |
| slr0710 | gdhA | glutamate dehydrogenase (NADP+) | 1.2 |
| slr0719 |  | unknown protein | 0.09 |
| slr0721 |  | malic enzyme | 1.67 |
| slr0722 |  | hypothetical protein | 0.11 |
| slr0723 |  | hypothetical protein | 0.09 |
| slr0729 |  | hypothetical protein | 1.58 |
| slr0730 |  | hypothetical protein | 0.78 |
| slr0731 |  | hypothetical protein | 2.86 |
| slr0737 | psaD | photosystem I subunit II | 1651.6 |
| slr0738 | trpE | anthranilate synthetase alpha-subunit | 0.67 |
| slr0740 |  | hypothetical protein | 0.54 |
| slr0743 |  | similar to N utilization substance protein | 3.38 |
| slr0744 | infB | translation initiation factor IF-2 | 0.36 |
| slr0748 |  | hypothetical protein | 0.28 |
| slr0752 |  | enolase | 25.39 |
| slr0756 | kaiA | circadian clock protein KaiA homolog | 1.11 |
| slr0757 | kaiB1 | circadian clock protein KaiB homolog | 2.95 |
| slr0758 | kaiC1 | circadian clock protein KaiC homolog | 1.32 |
| slr0765 |  | hypothetical protein | 1.01 |
| slr0769 |  | hypothetical protein | 0.17 |
| slr0773 |  | hypothetical protein | 0.82 |
| slr0774 | secD | protein-export membrane protein SecD | 0.15 |
| slr0775 | secF | protein-export membrane protein SecF | 0.11 |
| slr0782 |  | putative flavin-containing monoamine oxidase | 0.49 |
| slr0783 | tpi | triosephosphate isomerase | 1.97 |
| slr0806 |  | hypothetical protein | 1.64 |
| slr0807 |  | probable o-sialoglycoprotein endopeptidase | 0.1 |
| slr0808 |  | 16S rRNA processing protein RimM homolog | 1.16 |
| slr0809 | rfbB | dTDP-glucose 4,6-dehydratase | 0.74 |
| slr0817 | menF | salicylate biosynthesis isochorismate synthase | 0.07 |
| slr0821 |  | hypothetical protein | 3.71 |
| slr0823 | ycf3 | photosystem I assembly related protein | 1.22 |
| slr0825 |  | probable peptidase | 0.43 |
| slr0833 | dnaB | replicative DNA helicase [Contains: Ssp dnaB intein] | 0.04 |
| slr0835 |  | MoxR protein homolog | 0.73 |
| slr0838 | purM | phosphoribosyl formylglycinamidine cyclo-ligase | 0.32 |
| slr0839 | hemH | ferrochelatase | 0.45 |
| slr0841 |  | periplasmic protein, function unknown | 0.65 |
| slr0842 |  | hypothetical protein | 0.08 |
| slr0844 | ndhF1 | NADH dehydrogenase subunit 5 | 0.46 |
| slr0848 |  | hypothetical protein | 13.81 |
| slr0854 | phrA | DNA photolyase | 0.21 |
| slr0861 | purT | glycinamide ribonucleotide transformylase | 0.36 |
| slr0863 |  | hypothetical protein | 0.14 |
| slr0869 |  | hypothetical protein | 0.12 |
| slr0872 |  | hypothetical protein | 0.09 |
| slr0875 |  | large-conductance mechanosensitive channel | 0.5 |
| slr0876 |  | hypothetical protein | 0.27 |
| slr0877 |  | glutamyl-tRNA(Gln) amidotransferase subunit A | 1.13 |
| slr0879 |  | glycine decarboxylase complex H-protein | 0.27 |
| slr0884 | gap1 | glyceraldehyde 3-phosphate dehydrogenase 1 (NAD+) | 0.86 |
| slr0886 |  | 3-oxoacyl-[acyl-carrier protein] reductase | 3.58 |
| slr0889 |  | hypothetical protein | 0.08 |
| slr0891 |  | N-acetylmuramoyl-L-alanine amidase | 1.07 |
| slr0898 | nirA | ferredoxin--nitrite reductase | 0.8 |
| slr0899 | cynS | cyanate lyase | 0.69 |
| slr0905 | bchE | Mg-protoporphyrin IX monomethyl ester oxidative cyclase | 0.06 |
| slr0906 | psbB | photosystem II core light harvesting protein | 51.74 |
| slr0907 |  | unknown protein | 2.28 |
| slr0909 |  | unknown protein | 7.3 |
| slr0912 |  | unknown protein | 0.26 |
| slr0920 |  | mutator MutT protein | 0.44 |
| slr0923 | ycf65 | hypothetical protein YCF65 | 25.24 |
| slr0924 |  | periplasmic protein, function unknown | 0.38 |
| slr0925 | ssb | single-stranded DNA-binding protein | 16.65 |
| slr0929 |  | chromosome partitioning protein, ParA family | 0.63 |
| slr0930 |  | hypothetical protein | 1.44 |
| slr0940 | crtQ-2 | zeta-carotene desaturase | 0.99 |
| slr0941 |  | hypothetical protein | 0.73 |
| slr0942 |  | alcohol dehydrogenase [NADP+] | 1.93 |
| slr0943 | fda | fructose-bisphosphate aldolase, class I | 1.19 |
| slr0946 | arsC | arsenate reductase | 0.6 |
| slr0947 | rpaB | response regulator for energy transfer from phycobilisomes to photosystems | 11.73 |
| slr0948 |  | hypothetical protein | 0.16 |
| slr0952 | fbpII | fructose-1,6-bisphosphatase | 0.64 |
| slr0953 |  | sucrose-phosphate phosphatase | 0.14 |
| slr0955 |  | probable tRNA/rRNA methyltransferase | 0.18 |
| slr0958 | cysS | cysteinyl-tRNA synthetase | 0.14 |
| slr0962 |  | unknown protein | 4.74 |
| slr0965 | dnaN | DNA polymerase III beta subunit | 0.38 |
| slr0969 | cobJ | precorrin methylase | 0.05 |
| slr0974 | infC | initiation factor IF-3 | 0.19 |
| slr0976 |  | hypothetical protein | 0.65 |
| slr0977 |  | ABC transporter, permease component | 0.12 |
| slr0982 |  | probable polysaccharide ABC transporter ATP binding subunit | 0.08 |
| slr0983 | rfbF | glucose-1-phosphate cytidylyltransferase | 1.94 |
| slr0984 | rfbG | CDP-glucose 4,6-dehydratase | 0.19 |
| slr0985 | rfbC | dTDP-4-dehydrorhamnose 3,5-epimerase | 0.39 |
| slr1019 |  | phenazine biosynthetic protein PhzF homolog | 0.11 |
| slr1020 | sqdB | sulfolipid biosynthesis protein SqdB | 4.46 |
| slr1022 | argD | N-acetylornithine aminotransferase | 1.51 |
| slr1028 |  | unknown protein | 0.01 |
| slr1030 | chlI | magnesium protoporphyrin IX chelatase subunit I | 0.18 |
| slr1031 | tyrS | tyrosyl tRNA synthetase | 0.25 |
| slr1034 | ycf41 | hypothetical protein YCF41 | 5.69 |
| slr1035 |  | hypothetical protein | 0.19 |
| slr1039 |  | hypothetical protein | 0.31 |
| slr1042 |  | two-component response regulator CheY subfamily | 0.86 |
| slr1044 | ctr1 | methyl-accepting chemotaxis protein, required for the biogenesis of thick pilli | 1.68 |
| slr1046 |  | putative TatA protein | 0.29 |
| slr1048 |  | hypothetical protein | 0.03 |
| slr1051 |  | enoyl-[acyl-carrier-protein] reductase | 2.06 |
| slr1053 |  | unknown protein | 0.26 |
| slr1055 | chlH | magnesium protoporphyrin IX chelatase subunit H | 0.02 |
| slr1063 |  | probable glycosyltransferase | 0.05 |
| slr1067 |  | UDP-glucose 4-epimerase | 0.31 |
| slr1070 |  | unknown protein | 0.11 |
| slr1073 |  | unknown protein | 0.09 |
| slr1076 |  | probable glycosyltransferase | 0.09 |
| slr1077 |  | probable glycosyltransferase | 0.09 |
| slr1083 |  | hypothetical protein | 0.18 |
| slr1087 |  | hypothetical protein | 0.1 |
| slr1096 |  | dihydrolipoamide dehydrogenase | 5.85 |
| slr1097 |  | hypothetical protein | 7.56 |
| slr1098 |  | hypothetical protein | 0.14 |
| slr1099 | ubiX | 3-octaprenyl-4-hydroxybenzoate carboxy-lyase | 0.37 |
| slr1100 |  | hypothetical protein | 0.26 |
| slr1101 |  | hypothetical protein | 1.29 |
| slr1102 |  | hypothetical protein | 1.04 |
| slr1103 |  | hypothetical protein | 1.08 |
| slr1104 |  | hypothetical protein | 1.06 |
| slr1105 |  | GTP-binding protein TypA/BipA homolog | 0.06 |
| slr1106 |  | prohibitin | 10.54 |
| slr1109 |  | similar to ankyrin | 0.08 |
| slr1113 |  | ATP-binding protein of ABC transporter | 0.2 |
| slr1114 |  | hypothetical protein | 0.12 |
| slr1116 |  | hypothetical protein | 0.42 |
| slr1123 |  | guanylate kinase | 0.54 |
| slr1124 |  | phosphoglycerate mutase | 0.84 |
| slr1127 |  | unknown protein | 0.13 |
| slr1128 |  | hypothetical protein | 54.59 |
| slr1129 | rne | ribonuclease E | 0.47 |
| slr1133 | argH | L-argininosuccinate lyase | 6.27 |
| slr1136 | ctaCI | cytochrome c oxidase subunit II | 0.2 |
| slr1137 | ctaDI | cytochrome c oxidase subunit I | 0.12 |
| slr1139 | trxA | thioredoxin | 0.32 |
| slr1140 |  | DegT/DnrJ/EryC1/StrS family protein | 0.99 |
| slr1142 |  | hypothetical protein | 0.4 |
| slr1143 |  | hypothetical protein | 0.1 |
| slr1149 |  | ATP-binding protein of ABC transporter | 0.06 |
| slr1159 | purD | glycinamide ribonucleotide synthetase | 0.62 |
| slr1160 |  | periplasmic protein, function unknown | 0.34 |
| slr1161 |  | hypothetical protein | 2.76 |
| slr1165 |  | sulfate adenylyltransferase | 6.75 |
| slr1166 |  | UDP-glucose:tetrahydrobiopterin glucosyltransferase | 0.2 |
| slr1171 | gpx1 | glutathione peroxidase-like NADPH peroxidase,glutathione peroxidase | 0.46 |
| slr1176 |  | glucose-1-phosphate adenylyltransferase | 69.01 |
| slr1178 |  | hypothetical protein | 0.46 |
| slr1179 |  | hypothetical protein | 0.68 |
| slr1185 | petC2 | cytochrome b6-f complex alternative iron-sulfur subunit (Rieske iron sulfur protein) | 0.2 |
| slr1186 |  | hypothetical protein | 0.23 |
| slr1188 |  | hypothetical protein | 0.3 |
| slr1192 |  | probable alcohol dehydrogenase | 0.94 |
| slr1194 |  | hypothetical protein | 0.82 |
| slr1195 |  | hypothetical protein | 0.79 |
| slr1198 |  | antioxidant protein | 18.77 |
| slr1199 | mutL | DNA mismatch repair protein MutL | 0.06 |
| slr1205 |  | similar to chlorobenzene dioxygenase, ferredoxin component | 0.66 |
| slr1206 |  | hypothetical protein | 0.16 |
| slr1208 |  | probable oxidoreductase | 1.14 |
| slr1211 | cobN | cobalt-chelatase subunit CobN | 0.03 |
| slr1214 |  | two-component response regulator PatA subfamily | 0.09 |
| slr1216 |  | Mg2+ transport protein | 0.36 |
| slr1218 | ycf39 | hypothetical protein YCF39 | 0.22 |
| slr1220 |  | hypothetical protein | 1.39 |
| slr1223 |  | hypothetical protein | 3.24 |
| slr1224 |  | ATP-binding protein of sugar ABC transporter | 2.01 |
| slr1225 |  | serine/threonine kinase | 0.07 |
| slr1226 | purC | phosphoribosyl aminoidazole succinocarboxamide synthetase | 1.36 |
| slr1227 |  | chloroplastic outer envelope membrane protein homolog | 0.08 |
| slr1232 |  | unknown protein | 0.4 |
| slr1233 |  | succinate dehydrogenase flavoprotein subunit | 0.6 |
| slr1238 | gshB | glutathione synthetase | 0.94 |
| slr1239 | pntA | pyridine nucleotide transhydrogenase alpha subunit | 0.84 |
| slr1247 |  | phosphate-binding periplasmic protein precursor (PBP) | 1.24 |
| slr1251 |  | peptidyl-prolyl cis-trans isomerase | 3.4 |
| slr1253 |  | unknown protein | 0.34 |
| slr1254 | pds | phytoene dehydrogenase (phytoene desaturase) | 0.38 |
| slr1256 | ureA | urease gamma subunit | 0.36 |
| slr1258 |  | unknown protein | 6.95 |
| slr1263 |  | hypothetical protein | 0.07 |
| slr1265 | rpoC1 | RNA polymerase gamma-subunit | 14.22 |
| slr1267 | ftsW | cell division protein FtsW | 0.09 |
| slr1269 | ggt | gamma-glutamyltranspeptidase | 0.14 |
| slr1270 |  | periplasmic protein, function unknown | 0.13 |
| slr1272 |  | probable porin; major outer membrane protein | 0.26 |
| slr1273 |  | hypothetical protein | 1.05 |
| slr1274 | pilM | probable fimbrial assembly protein PilM, required for motility | 2.89 |
| slr1275 |  | hypothetical protein | 1.27 |
| slr1276 |  | hypothetical protein | 2.72 |
| slr1277 |  | pilus assembly protein homologous to general secretion pathway protein D | 0.18 |
| slr1278 | ycf62 | hypothetical protein YCF62 | 0.1 |
| slr1279 | ndhC | NADH dehydrogenase subunit 3 | 0.58 |
| slr1280 | ndhK | NADH dehydrogenase subunit NdhK | 143.77 |
| slr1281 | ndhJ | NADH dehydrogenase subunit I | 42.44 |
| slr1282 |  | putative transposase [ISY508b: 1877114 - 1878081] | 0.56 |
| slr1287 |  | hypothetical protein | 1.56 |
| slr1289 | icd | isocitrate dehydrogenase (NADP+) | 1.44 |
| slr1290 |  | hypothetical protein | 0.18 |
| slr1295 | futA1 | iron transport system substrate-binding protein | 19.97 |
| slr1299 |  | UDP-glucose dehydrogenase | 0.4 |
| slr1301 |  | hypothetical protein | 4.41 |
| slr1302 | cupB | protein involved in constitutive low affinity CO2 uptake | 0.08 |
| slr1306 |  | hypothetical protein | 0.07 |
| slr1311 | psbA2 | photosystem II D1 protein | 18.23 |
| slr1312 |  | arginine decarboxylase | 0.05 |
| slr1322 | tldD | putative modulator of DNA gyrase; TldD | 0.97 |
| slr1329 | atpB | ATP synthase beta subunit | 114 |
| slr1330 | atpE | ATP synthase epsilon chain of CF(1) | 0.88 |
| slr1331 |  | periplasmic processing protease | 0.14 |
| slr1334 |  | phosphoglucomutase/phosphomannomutase | 0.3 |
| slr1338 |  | hypothetical protein | 0.91 |
| slr1342 |  | hypothetical protein | 2.23 |
| slr1348 | cysE | serine acetyltransferase | 0.14 |
| slr1349 |  | glucose-6-phosphate isomerase | 40.54 |
| slr1351 | murF | UDP-N-acetylmuramoylalanyl-D-glutamyl-2 6-diaminopimelate--D-alanyl-D-alanine ligase | 2.16 |
| slr1356 | rps1a | 30S ribosomal protein S1 | 15.88 |
| slr1362 |  | hypothetical protein | 1.52 |
| slr1363 |  | hypothetical protein | 0.12 |
| slr1367 |  | glycogen phosphorylase | 8.77 |
| slr1379 | cydA | quinol oxidase subunit I | 0.14 |
| slr1383 |  | unknown protein | 0.68 |
| slr1385 |  | unknown protein | 0.22 |
| slr1390 | ftsH | cell division protein FtsH | 0.15 |
| slr1396 |  | unknown protein | 0.16 |
| slr1403 |  | unknown protein | 0.01 |
| slr1406 |  | periplasmic protein, function unknown | 4.88 |
| slr1407 |  | unknown protein | 0.24 |
| slr1409 |  | periplasmic WD-repeat protein | 1.58 |
| slr1410 |  | periplasmic WD-repeat protein | 1.79 |
| slr1411 |  | hypothetical protein | 0.74 |
| slr1414 | hik11 | two-component sensor histidine kinase | 0.24 |
| slr1415 |  | hypothetical protein | 0.09 |
| slr1417 | ycf57 | hypothetical protein YCF57 | 0.3 |
| slr1418 | pyrD | dihydroorotate dehydrogenase | 0.09 |
| slr1423 | murC | UDP-N-acetylmuramate-alanine ligase | 0.84 |
| slr1424 |  | UDP-N-acetylenolpyruvoylglucosamine reductase | 0.33 |
| slr1428 |  | hypothetical protein | 0.05 |
| slr1434 | pntB | pyridine nucleotide transhydrogenase beta subunit | 1.75 |
| slr1436 |  | unknown protein | 0.12 |
| slr1438 |  | hypothetical protein | 0.65 |
| slr1459 | apcF | phycobilisome core component | 133.47 |
| slr1463 | fus | elongation factor EF-G | 26.53 |
| slr1469 | rnpA | protein subunit of ribonuclease P (RNase P) | 0.27 |
| slr1470 |  | hypothetical protein | 0.78 |
| slr1471 |  | hypothetical protein | 1.08 |
| slr1476 | pyrB | aspartate carbamoyltransferase | 1.66 |
| slr1478 |  | hypothetical protein | 0.11 |
| slr1490 |  | ferrichrome-iron receptor | 0.04 |
| slr1503 |  | hypothetical protein | 0.4 |
| slr1506 |  | hypothetical protein | 0.83 |
| slr1510 | plsX | fatty acid/phospholipid synthesis protein PlsX | 0.41 |
| slr1511 | fabH | 3-oxoacyl-[acyl-carrier-protein] synthase III | 0.11 |
| slr1512 | sbtA | sodium-dependent bicarbonate transporter | 26.93 |
| slr1513 |  | periplasmic protein, function unknown | 192.47 |
| slr1515 | ictB | putative membrane protein required for bicarbonate uptake | 0.14 |
| slr1516 | sodB | superoxide dismutase | 6.95 |
| slr1517 | leuB | 3-isopropylmalate dehydrogenase | 3.04 |
| slr1519 |  | hypothetical protein | 1.07 |
| slr1523 |  | putative transposase | 0.14 |
| slr1531 | ffh | signal recognition particle protein | 0.07 |
| slr1533 |  | hypothetical protein | 0.31 |
| slr1534 |  | hypothetical protein | 0.22 |
| slr1536 | recQ | ATP-dependent DNA helicase RecQ | 0.07 |
| slr1537 |  | unknown protein | 0.6 |
| slr1540 |  | mRNA-binding protein | 0.92 |
| slr1541 |  | hypothetical protein | 1.58 |
| slr1545 | sigG | RNA polymerase ECF-type (group 3) sigma-E factor | 0.15 |
| slr1547 |  | hypothetical protein | 0.1 |
| slr1550 | lysS | lysyl-tRNA synthetase | 0.47 |
| slr1552 |  | unknown protein | 0.04 |
| slr1556 |  | 2-hydroxyacid dehydrogenase homolog | 0.62 |
| slr1559 | aroE | shikimate 5-dehydrogenase | 0.7 |
| slr1560 | hisS | histidyl tRNA synthetase | 1.49 |
| slr1562 |  | glutaredoxin | 2.44 |
| slr1563 |  | hypothetical protein | 0.11 |
| slr1571 |  | unknown protein | 0.15 |
| slr1573 |  | hypothetical protein | 0.53 |
| slr1577 |  | hypothetical protein | 0.21 |
| slr1579 |  | hypothetical protein | 0.12 |
| slr1585 |  | putative transposase [ISY508c(partial copy): 3405449 - 3406337] | 0.26 |
| slr1588 |  | two-component transcription regulator | 0.16 |
| slr1590 |  | hypothetical protein | 2.98 |
| slr1591 |  | hypothetical protein | 0.09 |
| slr1596 | pxcA | a protein in the cytoplasmic membrane involved in light-induced proton extrusion. | 0.07 |
| slr1600 |  | hypothetical protein | 2.27 |
| slr1603 |  | hypothetical protein | 0.32 |
| slr1604 | ftsH | cell division protein FtsH | 6.52 |
| slr1609 |  | long-chain-fatty-acid CoA ligase | 0.55 |
| slr1612 |  | hypothetical protein | 0.38 |
| slr1613 |  | hypothetical protein | 0.17 |
| slr1615 | rfbE | perosamine synthetase | 0.27 |
| slr1616 |  | unknown protein | 0.2 |
| slr1617 |  | similar to UDP-glucose 4-epimerase | 6.43 |
| slr1619 |  | hypothetical protein | 4.77 |
| slr1622 | ppa | soluble inorganic pyrophosphatase | 5.15 |
| slr1623 |  | hypothetical protein | 91.94 |
| slr1624 |  | hypothetical protein | 1.53 |
| slr1626 |  | dihydroneopterin aldolase | 0.69 |
| slr1634 |  | hypothetical protein | 0.58 |
| slr1639 |  | SsrA-binding protein | 0.21 |
| slr1641 | clpB1 | ClpB protein | 3.55 |
| slr1643 | petH | ferredoxin-NADP oxidoreductase | 86.69 |
| slr1644 |  | hypothetical protein | 0.12 |
| slr1645 | psb27 | photosystem II 11 kD protein | 53.89 |
| slr1649 |  | hypothetical protein | 6.71 |
| slr1651 |  | ABC transporter ATP-binding protein | 0.05 |
| slr1655 | psaL | photosystem I subunit XI | 27.03 |
| slr1658 |  | unknown protein | 0.93 |
| slr1659 |  | hypothetical protein | 6.07 |
| slr1665 | dapF | diaminopimelate epimerase | 0.12 |
| slr1666 |  | pleiotropic regulatory protein homolog | 0.73 |
| slr1668 |  | periplasmic protein, function unknown (target gene of sycrp1) | 0.12 |
| slr1670 |  | unknown protein | 0.04 |
| slr1678 | rpl21 | 50S ribosomal protein L21 | 13.37 |
| slr1679 |  | hypothetical protein | 0.13 |
| slr1681 |  | unknown protein | 0.11 |
| slr1691 |  | glutamine-dependent NAD(+) synthetase | 0.12 |
| slr1693 |  | two-component response regulator PatA subfamily | 1.01 |
| slr1694 |  | expression activator appA homolog | 3.17 |
| slr1702 |  | hypothetical protein | 0.7 |
| slr1703 | serS | seryl-tRNA synthetase | 1.35 |
| slr1706 |  | dihydroflavonol 4-reductase | 0.1 |
| slr1712 |  | hypothetical protein | 0.38 |
| slr1718 |  | hypothetical protein | 1.17 |
| slr1719 |  | DrgA protein homolog | 18.05 |
| slr1720 | aspS | aspartyl-tRNA synthetase | 3.37 |
| slr1721 |  | hypothetical protein | 0.39 |
| slr1722 |  | inosine-5'-monophosphate dehydrogenase | 9.23 |
| slr1724 |  | hypothetical protein | 1.76 |
| slr1727 |  | Na+/H+ antiporter | 0.55 |
| slr1729 | kdpB | potassium-transporting P-type ATPase B chain | 0.2 |
| slr1732 |  | hypothetical protein | 0.69 |
| slr1734 | opcA | glucose 6-phosphate dehydrogenase assembly protein | 2.94 |
| slr1735 | bgtA | ATP-binding subunit of the ABC-type Bgt permease for basic amino acids and glutamine | 3.42 |
| slr1739 | psb28-2 | photosystem II 13 kDa protein homolog | 0.91 |
| slr1740 |  | oligopeptide binding protein of ABC transporter | 0.3 |
| slr1742 |  | probable cobyric acid synthase | 0.26 |
| slr1743 | ndbB | type 2 NADH dehydrogenase NdbB | 0.43 |
| slr1751 |  | periplasmic carboxyl-terminal protease | 3.48 |
| slr1755 |  | NAD+ dependent glycerol-3-phosphate dehydrogenase | 0.23 |
| slr1756 | glnA | glutamate--ammonia ligase | 92.61 |
| slr1761 |  | FKBP-type peptidyl-prolyl cis-trans isomerase, periplasmic protein | 2.65 |
| slr1763 |  | probable methyltransferase | 2.05 |
| slr1768 |  | unknown protein | 11.84 |
| slr1771 |  | unknown protein | 0.06 |
| slr1772 |  | probable hydrolase, periplasmic protein | 0.63 |
| slr1773 |  | unknown protein | 0.45 |
| slr1777 | chlD | magnesium protoporphyrin IX chelatase subunit D | 0.05 |
| slr1778 |  | unknown protein | 0.19 |
| slr1779 | pdxJ | pyridoxal phosphate biosynthetic protein PdxJ | 2.55 |
| slr1780 | ycf54 | hypothetical protein YCF54 | 3.05 |
| slr1783 | ycf29 | two-component response regulator NarL subfamily | 1.33 |
| slr1784 | bvdR | biliverdin reductase | 0.2 |
| slr1788 |  | unknown protein | 0.09 |
| slr1790 |  | hypothetical protein | 0.32 |
| slr1791 | cysH | phosphoadenosine phosphosulfate reductase | 0.26 |
| slr1793 |  | transaldolase | 17.83 |
| slr1794 |  | probable anion transporting ATPase | 2.26 |
| slr1803 |  | adenine-specific DNA methylase | 0.1 |
| slr1809 |  | unknown protein | 0.13 |
| slr1814 |  | hypothetical protein | 0.46 |
| slr1816 |  | hypothetical protein | 0.31 |
| slr1818 |  | hypothetical protein | 0.93 |
| slr1828 | petF | ferredoxin, petF-like protein | 0.35 |
| slr1829 | phaE | putative poly(3-hydroxyalkanoate) synthase component | 0.4 |
| slr1830 | phaC | poly(3-hydroxyalkanoate) synthase | 0.18 |
| slr1834 | psaA | P700 apoprotein subunit Ia | 28.76 |
| slr1835 | psaB | P700 apoprotein subunit Ib | 25.16 |
| slr1837 |  | two-component system response regulator OmpR subfamily | 0.47 |
| slr1838 | ccmK3 | carbon dioxide concentrating mechanism protein CcmK homolog 3, putative carboxysome assembly protein | 0.36 |
| slr1839 | ccmK4 | carbon dioxide concentrating mechanism protein CcmK homolog 4, putative carboxysome assembly protein | 15.37 |
| slr1841 |  | probable porin; major outer membrane protein | 3.84 |
| slr1842 | cysK | cysteine synthase | 1.27 |
| slr1843 | zwf | glucose 6-phosphate dehydrogenase | 40.78 |
| slr1846 | ycf64 | hypothetical protein YCF64 | 0.66 |
| slr1847 |  | hypothetical protein | 4.8 |
| slr1848 | hisD | histidinol dehydrogenase | 2.99 |
| slr1852 |  | unknown protein | 16.91 |
| slr1853 |  | carboxymuconolactone decarboxylase | 1.39 |
| slr1854 |  | unknown protein | 9.11 |
| slr1855 |  | unknown protein | 9.81 |
| slr1856 |  | phosphoprotein substrate of icfG gene cluster | 1.7 |
| slr1857 |  | isoamylase | 0.33 |
| slr1859 |  | anti-sigma f factor antagonist | 2.8 |
| slr1860 | icfG | carbon metabolisms regulatory protein IcfG | 0.26 |
| slr1861 |  | probable sigma regulatory factor | 0.25 |
| slr1867 | trpD | anthranilate phosphoribosyltransferase | 0.44 |
| slr1870 |  | hypothetical protein | 0.14 |
| slr1874 |  | D-alanine--D-alanine ligase | 0.2 |
| slr1877 |  | 2-hydroxyhepta-2,4-diene-1,7-dioate isomerase | 0.73 |
| slr1879 | cobI | precorrin-2 methyltransferase | 0.14 |
| slr1881 | natE | ATP-binding subunit of the ABC-type Nat permease for neutral amino acids | 0.15 |
| slr1884 | trpS | tryptophanyl-tRNA synthetase | 0.54 |
| slr1887 | hemC | porphobilinogen deaminase (hydroxymethylbilane synthase, preuroporphyrinogen synthase) | 2.9 |
| slr1888 |  | 4-hydroxybutyrate coenzyme A transferase. | 0.1 |
| slr1890 |  | bacterioferritin | 18.88 |
| slr1894 |  | probable DNA-binding stress protein | 44.21 |
| slr1897 |  | periplasmic sugar-binding protein of ABC transporter | 0.25 |
| slr1898 | argB | N-acetylglutamate kinase | 0.37 |
| slr1900 |  | hypothetical protein | 1.31 |
| slr1901 |  | ATP-binding protein of ABC transporter | 0.1 |
| slr1907 |  | hypothetical protein | 0.4 |
| slr1908 |  | probable porin; major outer membrane protein | 0.24 |
| slr1909 |  | two-component response regulator NarL subfamily | 3.81 |
| slr1915 |  | hypothetical protein | 0.32 |
| slr1916 |  | probable esterase | 0.12 |
| slr1923 |  | hypothetical protein | 0.33 |
| slr1924 |  | D-alanyl-D-alanine carboxypeptidase, periplasmic protein | 0.35 |
| slr1926 |  | hypothetical protein | 0.66 |
| slr1927 |  | hypothetical protein | 0.16 |
| slr1934 |  | pyruvate dehydrogenase E1 component, alpha subunit | 11.47 |
| slr1938 |  | putative translation initiation factor EIF-2b subunit 1 | 1.6 |
| slr1940 |  | periplasmic protein, function unknown | 2.74 |
| slr1942 | kaiC3 | circadian clock protein KaiC homolog | 1.26 |
| slr1944 |  | periplasmic protein, function unknown | 0.45 |
| slr1945 |  | 2,3-bisphosphoglycerate-independent phosphoglycerate mutase | 0.39 |
| slr1949 |  | hypothetical protein | 1.16 |
| slr1950 | ctaA | copper-transporting P-type ATPase CtaA | 0.04 |
| slr1951 |  | hypothetical protein | 0.71 |
| slr1958 |  | unknown protein | 1.52 |
| slr1962 |  | probable extracellular solute-binding protein | 0.28 |
| slr1963 |  | water-soluble carotenoid protein | 67.83 |
| slr1970 |  | hypothetical protein | 1.11 |
| slr1972 | ycf81 | hypothetical protein YCF81 | 0.3 |
| slr1975 |  | N-acylglucosamine 2-epimerase | 0.44 |
| slr1982 |  | two-component response regulator CheY subfamily | 0.66 |
| slr1983 |  | two-component hybrid sensor and regulator | 0.06 |
| slr1984 | rps1b | nucleic acid-binding protein, 30S ribosomal protein S1 homolog | 5.78 |
| slr1986 | apcB | allophycocyanin beta subunit | 10829 |
| slr1992 | gpx2 | glutathione peroxidase-like NADPH peroxidase | 2.74 |
| slr1993 | phaA | PHA-specific beta-ketothiolase | 0.76 |
| slr1994 | phaB | PHA-specific acetoacetyl-CoA reductase | 2.62 |
| slr2000 |  | hypothetical protein | 0.22 |
| slr2001 | cphB | cyanophycinase | 0.93 |
| slr2002 | cphA | cyanophycin synthetase | 4.68 |
| slr2004 |  | periplasmic protein, function unknown | 0.31 |
| slr2011 |  | hypothetical protein | 1.11 |
| slr2015 | pilA9 | type 4 pilin-like protein, essential for motility | 0.47 |
| slr2018 |  | unknown protein | 1.03 |
| slr2023 | fabD | malonyl coenzyme A-acyl carrier protein transacylase | 0.4 |
| slr2024 |  | two-component response regulator CheY subfamily | 0.38 |
| slr2025 |  | hypothetical protein | 1.79 |
| slr2032 | ycf23 | hypothetical protein YCF23 | 5.48 |
| slr2033 | rubA | membrane-associated rubredoxin, essential for photosystem I assembly | 10.37 |
| slr2034 | ycf48 | putative homolog of plant HCF136, which is essential for stability or assembly of photosystem II | 4.65 |
| slr2043 |  | zinc transport system substrate-binding protein | 0.1 |
| slr2049 | ycf58 | hypothetical protein YCF58 | 1.11 |
| slr2051 | cpcG1 | phycobilisome rod-core linker polypeptide | 989.61 |
| slr2053 |  | putative hydrolase | 0.12 |
| slr2058 | topA | DNA topoisomerase I | 0.24 |
| slr2059 |  | iron-sulfur cluster binding protein homolog | 1.14 |
| slr2060 |  | hypothetical protein | 0.14 |
| slr2067 | apcA | allophycocyanin alpha subunit | 23067 |
| slr2070 |  | hypothetical protein | 1.47 |
| slr2072 | ilvA | L-threonine deaminase | 2.74 |
| slr2075 | groES | 10kD chaperonin | 3.53 |
| slr2076 | groEL1 | 60kD chaperonin | 110.49 |
| slr2084 |  | hypothetical protein | 0.3 |
| slr2088 | ilvG | acetohydroxy acid synthase | 1.48 |
| slr2089 | shc | squalene-hopene-cyclase | 0.05 |
| slr2094 | fbpI | fructose-1,6-/sedoheptulose-1,7-bisphosphatase | 143.81 |
| slr2100 |  | two-component response regulator | 0.09 |
| slr2102 | ftsY | cell division protein FtsY | 0.36 |
| slr2105 |  | hypothetical protein | 0.84 |
| slr2122 |  | hypothetical protein | 0.31 |
| slr2123 |  | similar to D-3-phosphoglycerate dehydrogenase | 0.73 |
| slr2124 |  | 3-oxoacyl-[acyl-carrier protein] reductase | 0.14 |
| slr2130 | aroB | 3-dehydroquinate synthase | 0.64 |
| slr2131 |  | RND multidrug efflux transporter | 1.18 |
| slr2132 |  | phosphotransacetylase | 0.64 |
| slr2136 |  | GcpE protein homolog | 1.8 |
| slr2141 |  | hypothetical protein | 0.1 |
| slr2143 |  | L-cysteine/cystine lyase | 0.18 |
| slr2144 |  | periplasmic protein, function unknown | 0.86 |
| slr5005 |  | hypothetical protein | 0.01 |
| slr5012 |  | hypothetical protein | 0.11 |
| slr5023 |  | hypothetical protein | 0.08 |
| slr5056 |  | probable glycosyltransferase | 0.07 |
| slr5088 |  | probable short-chain dehydrogenase | 0.14 |
| slr5110 |  | unknown protein | 0.5 |
| slr6001 |  | two-component hybrid sensor and regulator | 0.08 |
| slr6012 |  | unknown protein | 1.41 |
| slr6013 |  | unknown protein | 0.24 |
| slr6043 |  | probable cation efflux system protein, czcA homolog | 0.06 |
| slr6050 |  | hypothetical protein | 0.06 |
| slr6095 |  | type I restriction-modification system, M subunit (fragment) | 0.42 |
| slr6096 |  | type I restriction-modification system, M subunit (fragment) | 0.38 |
| slr6100 |  | hypothetical protein | 0.32 |
| slr7011 |  | unknown protein | 0.21 |
| slr7012 |  | hypothetical protein | 2.5 |
| slr7024 |  | hypothetical protein | 0.2 |
| slr7061 |  | unknown protein | 0.08 |
| slr7068 |  | hypothetical protein | 0.09 |
| slr8030 |  | hypothetical protein | 0.05 |
| slr8038 |  | WD-repeat protein | 0.03 |
| slr8044 |  | unknown protein | 0.07 |
| sml0006 | rpl36 | 50S ribosomal protein L36 | 10.45 |
| sml0007 | psbY | photosystem II protein Y | 5.42 |
| sml0008 | psaJ | photosystem I subunit IX | 6.86 |
| smr0005 | psaM | photosystem I subunit XII | 1.42 |
| smr0006 | psbF | cytochrome b559 b subunit | 4.5 |
| smr0007 | psbL | photosystem II PsbL protein | 14.11 |
| ssl0020 | petF | ferredoxin I, essential for growth | 0.89 |
| ssl0242 |  | hypothetical protein | 0.45 |
| ssl0294 |  | hypothetical protein | 1.04 |
| ssl0352 |  | hypothetical protein | 61.89 |
| ssl0546 | minE | septum site-determining protein MinE | 5.21 |
| ssl0563 | psaC | photosystem I subunit VII | 3574.6 |
| ssl0601 | rps21 | 30S ribosomal protein S21 | 13.72 |
| ssl0707 | glnB | nitrogen regulatory protein P-II | 181.41 |
| ssl0788 |  | hypothetical protein | 0.76 |
| ssl1046 |  | hypothetical protein | 6.02 |
| ssl1426 | rpl35 | 50S ribosomal protein L35 | 4.09 |
| ssl1498 |  | hypothetical protein | 1.32 |
| ssl1533 |  | unknown protein | 2.48 |
| ssl1690 |  | hypothetical protein | 46.98 |
| ssl1707 |  | hypothetical protein | 0.43 |
| ssl1784 | rps15 | 30S ribosomal protein S15 | 36.94 |
| ssl1972 |  | hypothetical protein | 1.37 |
| ssl2084 | acpP | acyl carrier protein | 0.96 |
| ssl2148 |  | hypothetical protein | 1.52 |
| ssl2233 | rps20 | 30S ribosomal protein S20 | 12.18 |
| ssl2296 |  | pterin-4a-carbinolamine dehydratase | 4.35 |
| ssl2501 |  | unknown protein | 0.38 |
| ssl2595 |  | hypothetical protein | 2.95 |
| ssl2598 | psbH | photosystem II PsbH protein | 21.9 |
| ssl2615 | atpH | ATP synthase C chain of CF(0) | 1.02 |
| ssl2667 | cnfU | an assembly factor for iron-sulfur culsters | 0.49 |
| ssl2874 |  | hypothetical protein | 19.41 |
| ssl3044 |  | probable ferredoxin | 2.81 |
| ssl3093 | cpcD | phycobilisome small rod linker polypeptide | 1069.4 |
| ssl3127 |  | similar to permease protein of ABC transporter | 0.5 |
| ssl3335 | secE | preprotein translocase SecE subunit | 0.9 |
| ssl3364 | cp12 | CP12 polypeptide | 3.54 |
| ssl3432 | rps19 | 30S ribosomal protein S19 | 41.51 |
| ssl3436 | rpl29 | 50S ribosomal protein L29 | 21.92 |
| ssl3437 | rps17 | 30S ribosomal protein S17 | 211.79 |
| ssl3445 | rpl31 | 50S ribosomal protein L31 | 3.01 |
| ssl3451 |  | hypothetical protein | 0.45 |
| ssl5100 |  | hypothetical protein | 0.51 |
| ssl7038 |  | hypothetical protein | 0.78 |
| ssl7045 |  | unknown protein | 0.55 |
| ssl7053 |  | hypothetical protein | 1.38 |
| ssl8005 |  | hypothetical protein | 0.37 |
| ssr0330 | ftrV | ferredoxin-thioredoxin reductase, variable chain | 7.18 |
| ssr0332 |  | hypothetical protein | 1.25 |
| ssr0390 | psaK1 | photosystem I reaction center subunit X | 0.47 |
| ssr0482 | rps16 | 30S ribosomal protein S16 | 407.3 |
| ssr0761 |  | hypothetical protein | 1.52 |
| ssr1391 |  | hypothetical protein | 0.37 |
| ssr1398 | rpl33 | 50S ribosomal protein L33 | 16.2 |
| ssr1399 | rps18 | 30S ribosomal protein S18 | 28.24 |
| ssr1480 | rbp2 | putative RNA-binding protein | 14.71 |
| ssr1528 |  | hypothetical protein | 41.06 |
| ssr1600 |  | similar to anti-sigma f factor antagonist | 22.27 |
| ssr1604 | rpl28 | 50S ribosomal protein L28 | 65.61 |
| ssr1765 |  | hypothetical protein | 0.42 |
| ssr1766 |  | hypothetical protein | 0.47 |
| ssr1789 | hliD | CAB/ELIP/HLIP-related protein HliD | 4.62 |
| ssr2061 |  | glutaredoxin | 2.73 |
| ssr2194 |  | unknown protein | 2.4 |
| ssr2754 |  | hypothetical protein | 0.42 |
| ssr2799 | rpl27 | 50S ribosomal protein L27 | 55.97 |
| ssr2803 |  | hypothetical protein | 0.4 |
| ssr2831 | psaE | photosystem I subunit IV | 132.14 |
| ssr2857 | atx1 | mercuric transport protein periplasmic component precursor | 1.86 |
| ssr2998 |  | hypothetical protein | 10.5 |
| ssr3383 | apcC | phycobilisome small core linker polypeptide | 371.26 |
| ssr3451 | psbE | cytochrome b559 alpha subunit | 21.76 |

For symbols and annotations, we followed the Synechocystis annotation of Cyanobase (http://genome.kazusa.or.jp/cyanobase/). The emPAI value of each protein showed a sum of emPAI values in all the gel slices from the BN-gel.
